# Association of Anorexia Nervosa With Risk of Cancer: A Systematic Review and Meta-analysis

**DOI:** 10.1001/jamanetworkopen.2019.5313

**Published:** 2019-06-07

**Authors:** Ferrán Catalá-López, Jaume Forés-Martos, Jane A. Driver, Matthew J. Page, Brian Hutton, Manuel Ridao, Adolfo Alonso-Arroyo, Diego Macías Saint-Gerons, Ricard Gènova-Maleras, José M. Valderas, Eduard Vieta, Alfonso Valencia, Rafael Tabarés-Seisdedos

**Affiliations:** 1 Department of Health Planning and Economics, National School of Public Health, Institute of Health Carlos III, Madrid, Spain; 2 Department of Medicine, University of Valencia, INCLIVA Health Research Institute, Centro de Investigación en Red de Salud Mental (CIBERSAM), Valencia, Spain; 3 Clinical Epidemiology Program, Ottawa Hospital Research Institute, Ottawa, Ontario, Canada; 4 Geriatric Research Education and Clinical Center, Veterans Affairs Boston Healthcare System, Boston, Massachusetts; 5 Division of Aging, Department of Medicine, Brigham and Women’s Hospital, Harvard Medical School, Boston, Massachusetts; 6 Department of Medical Oncology, Dana-Farber Cancer Institute, Boston, Massachusetts; 7 School of Public Health and Preventive Medicine, Monash University, Melbourne, Australia; 8 School of Epidemiology and Public Health, University of Ottawa, Ottawa, Ontario, Canada; 9 Instituto Aragonés de Ciencias de la Salud, Red de Investigación en Servicios de Salud en Enfermedades Crónicas, Zaragoza, Spain; 10 Department of History of Science and Documentation, University of Valencia, Valencia, Spain; 11 Unidad de Información e Investigación Social y Sanitaria, University of Valencia, Spanish National Research Council, Valencia, Spain; 12 Directorate General for Public Health, Regional Health Council, Madrid, Spain; 13 Health Services and Policy Research Group, Exeter Collaboration for Academic Primary Care, University of Exeter Medical School, University of Exeter, Exeter, United Kingdom; 14 Hospital Clínic, Universitat de Barcelona, Institut d’Investigacions Biomèdiques August Pi i Sunyer, Centro de Investigación en Red de Salud Mental (CIBERSAM), Barcelona, Spain; 15 Life Sciences Department, Barcelona Supercomputing Center, Barcelona, Spain

## Abstract

**Importance:**

Anorexia nervosa is recognized as an important cause of morbidity in young people. However, the risk of cancer in people with anorexia nervosa remains uncertain.

**Objective:**

To evaluate the association of anorexia nervosa with the risk of developing or dying of cancer.

**Data Sources:**

MEDLINE, Scopus, Embase, and Web of Science from database inception to January 9, 2019.

**Study Selection:**

Published observational studies in humans examining the risk of cancer in people with anorexia nervosa compared with the general population or those without anorexia nervosa. Studies needed to report incidence or mortality rate ratios (RRs).

**Data Extraction and Synthesis:**

Screening, data extraction, and methodological quality assessment were performed by at least 2 researchers independently. A random-effects model was used to synthesize individual studies. Heterogeneity (*I^2^*) was assessed and 95% prediction intervals (PIs) were calculated.

**Main Outcomes and Measures:**

All cancer incidence and cancer mortality associated with anorexia nervosa. Secondary outcomes were site-specific cancer incidence and mortality.

**Results:**

Seven cohort studies published in 10 articles (42 602 participants with anorexia nervosa) were included. Anorexia nervosa was not associated with risk of developing any cancer (4 studies in women; RR, 0.97; 95% CI, 0.89-1.06; *P* = .53; *I*^2^, 0%; 95% PI, 0.80-1.18; moderate confidence). Anorexia nervosa was associated with decreased breast cancer incidence (5 studies in women; RR, 0.60; 95% CI, 0.50-0.80; *P* < .001; *I*^2^, 0%; 95% PI, 0.44-0.83; high confidence). Conversely, anorexia nervosa was associated with increased risk of developing lung cancer (3 studies in women; RR, 1.50; 95% CI, 1.06-2.12; *P* = .001; *I*^2^, 0%; 95% PI, 0.19-16.46; low confidence) and esophageal cancer (2 studies in women; RR, 6.10; 95% CI, 2.30-16.18; *P* < .001; *I*^2^, 0%; low confidence).

**Conclusions and Relevance:**

Among people with anorexia nervosa, risk of developing cancer did not differ compared with the general population, but a significantly reduced risk of breast cancer was observed. Understanding the mechanisms underlying these associations could have important preventive potential.

## Key Points

**Question:**

Are people with anorexia nervosa at a higher risk of developing or dying of cancer compared with those without anorexia and the general population?

**Findings:**

In this systematic review and meta-analysis of 7 cohort studies including more than 42 000 participants with anorexia nervosa, there was no association of anorexia nervosa with overall cancer incidence or mortality. Anorexia nervosa was inversely associated with breast cancer incidence but positively associated with risk of developing lung and esophageal cancer.

**Meaning:**

There was no association of anorexia nervosa with risk of cancer overall and few associations of anorexia nervosa with risk of site-specific cancer.

## Teaser

This systematic review and meta-analysis evaluates the association of anorexia nervosa with the cancer incidence and mortality among study populations with anorexia nervosa compared with the general population or those without anorexia nervosa.

## Introduction

Almost 3.4 million young people throughout the world have anorexia nervosa.^1^ Anorexia nervosa is characterized by a severe restriction of caloric intake, extremely low body weight, fear of gaining weight or of becoming fat, and disturbance of body image. Anorexia nervosa is more commonly reported by young women and girls, but the disorder is increasingly being reported by boys, men, and women older than 40 years. Genetic factors and hormonal changes may influence risk, psychosocial and interpersonal factors can trigger onset, and changes in molecular pathways and cellular networks in the brain are associated with the illness and its comorbidities.^2,3,4^

Cancer is the second leading cause of death worldwide,^5,6^ with more than 9.6 million deaths in 2017.^6^ Multiple studies and meta-analyses suggest that excess body weight is a risk factor of several cancers.^7,8,9^ For example, a recent umbrella review^8^ found evidence of an association of body mass index (BMI) with cancers of digestive organs, hormone-related cancers, uterine cancer, kidney cancer, and multiple myeloma and also of adiposity with the risk of colorectal cancer, gallbladder cancer, gastric cancer, ovarian cancer, and multiple myeloma. The underlying mechanisms of the association of excess body weight with cancer are complex and are not yet fully understood.

Research on how reducing body weight might lower the risk of developing cancer is limited, to our knowledge. Energy restriction (or calorie restriction) has been found to be protective against the development of cancer in experimental animal studies.^10^ In addition, certain calorie-limited diets or fasting could influence the risk of developing cancer. Anorexia nervosa, an excessive form of calorie restriction associated with pathological weight loss, has been proposed as a biomarker of energy restriction in humans.^11,12,13^ Several studies have evaluated whether there is a reduction in cancer development among people with anorexia nervosa.^12,13,14^ However, to our knowledge, there are no systematic reviews and meta-analyses investigating the strength of the evidence of the potential associations of anorexia nervosa with cancer. In this study, we aimed to evaluate the association of anorexia nervosa with the risk of developing or dying of cancer.

## Methods

We followed the current methods recommendations for systematic reviews^15,16^ and developed a protocol (PROSPERO registration number: CRD42017067462)^17^ to comply with the Preferred Reporting Items for Systematic Reviews and Meta-analyses (PRISMA) reporting guideline statement for reporting this study. We also reported this study in accordance with the Meta-analysis of Observational Studies in Epidemiology (MOOSE) reporting guideline. Our methods are briefly described here and explained in more detail in the published protocol^17^ and in eTables 1-7 in the Supplement.

### Search Strategy and Selection Criteria

One of us (A.A.-A.) searched MEDLINE, Scopus, Embase, and the Web of Science to identify all relevant observational studies in humans that examined the association of anorexia nervosa with the risk of cancer published from database inception to January 9, 2019, without language restrictions. The main search strategy for MEDLINE is presented in eTable 3 in the Supplement. This search strategy was adapted to fit with other databases. To supplement these searches, references of all relevant primary studies and review articles were also screened to identify additional data sources.

To be included, primary studies had to be observational (ie, cohort or case-control) studies of people with anorexia nervosa (according to standard operationalized diagnostic criteria, ie, *International Classification of Diseases, Ninth Revision* [*ICD-9*] codes 307.1 or 307.54 or *ICD-10* codes F50.0-F50.1) and report the incidence or mortality rate ratios (RRs) for the risk of cancer in patients with anorexia nervosa compared with the general population or those without anorexia nervosa or have enough data (ie, number of cases and sample size, observed and expected cases) to compute these estimates. We excluded studies in which anorexia nervosa was not the exposure of interest and in which cancer was not reported as the outcome. Studies not presenting study-specific data or sufficient data for an outcome measure to be calculated were also excluded. We excluded case reports, case series, in vitro studies, and animal studies.

Two of us (F.C.-L. and J.F.-M.) independently screened the titles and abstracts of articles retrieved from the literature search, and the full-texts of potentially eligible articles were obtained and further assessed for final inclusion. Disagreements were resolved through discussions until a consensus was reached.

### Outcomes

The primary outcomes were all cancer incidences and cancer mortalities (all malignant neoplasms; *ICD-9* codes 140-209; *ICD-10* codes C00-C97). Given the varied biology of cancers, the risk of incident site-specific cancers and the risk of fatal site-specific cancers were evaluated as secondary outcomes (eTable 1 in the Supplement).

### Data Extraction and Quality Assessment

Two of us (F.C.-L. and J.F.-M.) independently extracted article data, including first author, publication year, period of recruitment, country, study design, setting, coverage, mean or median age (or age range), proportion of female participants, race/ethnicity, parity status, profile of tobacco smoking, ascertainment of cancer diagnosis and diagnostic criteria, consideration of confounding factors, the number of participants with cancer, and maximally adjusted RR estimates with 95% CIs from the included studies. For duplicate study publications, we considered only the report with the most informative and complete data. When relevant outcome data were not available, we directly contacted the corresponding author of the study to request the information. Two pairs of us (J.F.-M., M.J.P., M.R., and/or D.M.S.-G.) independently undertook methodological quality assessment of included studies using the Newcastle-Ottawa scale^18^ and allocated stars for adherence to the prespecified criteria. This scale ranges from 0 stars (lowest quality) to 9 stars (highest quality) and judges each study regarding selection of study groups, comparability, and ascertainment of the outcome. We considered studies with 0 to 3 stars to represent high risk of bias; 4 to 6 stars, moderate risk of bias; and 7 to 9 stars, low risk of bias. Discrepant scores were resolved by discussion and consensus among us.

### Statistical Analysis

To measure the association of anorexia nervosa with cancer, we performed meta-analysis using the inverse–variance weighted random-effects model^19^ to pool weighted RRs of cancer risk estimates. Random-effects meta-analysis produces a more generalizable result by considering both within-study and between-study variation by incorporating the heterogeneity of effects into overall analyses. We assessed heterogeneity between studies using the *P* value of Cochran *Q* test^20^ and the *I*^2^ statistic (with 95% CIs).^21^ The *I*^2^ statistic ranges from 0% to 100%, with values of 0% to 25% indicating low heterogeneity and 75% to 100% indicating high heterogeneity.^15^ In comparisons that included 3 or more studies, we also calculated the 95% prediction intervals (PIs), which further account for heterogeneity between studies and indicate the uncertainty for the effect that would be expected in a new study examining that same association.^22^ To assess the robustness of pooled results and explore possible reasons for heterogeneity, prespecified subgroup analyses were performed according to sex (female or male), and cancer types according to relationship with smoking (smoking-related cancer sites or other cancer sites) or sex hormones (cancers occurring in hormone-sensitive tissues, such as breast, ovary, uterus, prostate, and colorectal, where sex hormones exert an important influence in cancer etiopathogenesis and progression, or other cancer sites).^17^ For the secondary outcome of breast cancer incidence, we conducted a specific analysis among women and girls based on parity status (parous or nulliparous women) and age at receiving first diagnosis of anorexia nervosa (prior to age 20 years or at 20 years or older).^13^ We performed a post hoc sensitivity analysis excluding studies that had possible geographical and temporal overlapping study samples (eg, 2 previously published studies in Denmark^12^ and Sweden^23,24,25^), considering only data from the study with the greatest person-years. *P* values were determined using the Woolf method. *P* values were 2-tailed, and statistical significance was set at less than .001. We conducted data analysis using the metan package in Stata statistical software version 15 (StataCorp).

With the publication of this article, the full data set will be freely available online in the Open Science Framework (https://osf.io/Y6WQN/). We applied a set of criteria to classify the credibility of the evidence from meta-analysis based on the Global Burden of Disease and World Cancer Research Fund criteria^26,27^ and GRADE system^28^ (eTable 7 in the Supplement).

## Results

### Relevant Literature Identified

**Figure 1.**
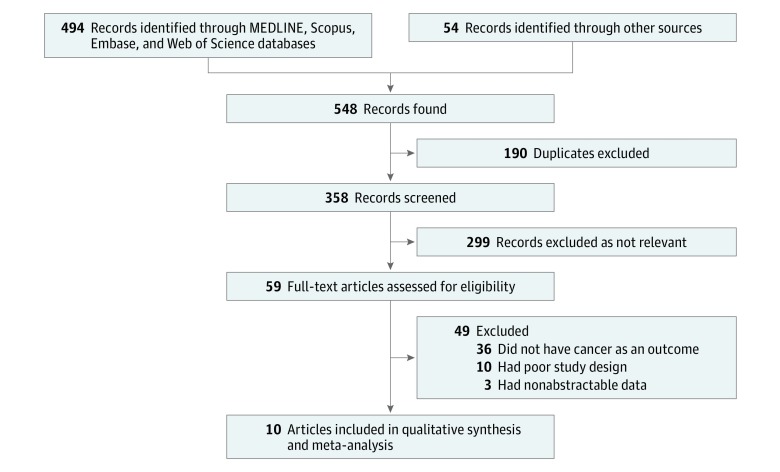
Flow Diagram of Study Selection

We screened 358 titles and abstracts, followed by 59 full-text articles (Figure 1). Seven cohort studies published in 10 articles,^12,13,23,24,25,29,30,31,32,33^ including 42 602 participants with anorexia nervosa, met our inclusion criteria.

### Description of Studies and Participants

Table 1 summarizes the characteristics of the included studies. All studies were published in English from 2001 through 2018. Five studies were from Europe (Denmark,^12,29,32^ Finland,^29,32^ Sweden,^13,23,24,25,29,32^ and the United Kingdom^31^) and 2 studies^30,33^ were from North America (the United States). Six studies were retrospective cohorts^12,13,23,24,25,29,30,31,32^ and 1 was a prospective cohort.^33^ Median (range) follow-up was 13.3 (5.4-27.1) years. The number of participants with anorexia nervosa varied from 275 to 24 332, and the number of cancer cases ranged from 2 to 389. The proportion of female participants varied from 60.5% to 100%.

**Table 1.** Characteristics of Included Studies

Source	Study Design (Country)	Setting; Coverage	Study Years (Follow-up, y)	No. of Participants with AN	Characteristics of Participants by Sex; Age; Race/Ethnicity; Parity Status	No. of Cancer Cases	Main Cancer Outcome	Site-Specific Cancer	Exposure	Outcome Definitions	Comparator	End Point Measure	Adjustment for Confounding Factors
Mellemkjaer et al,^12^ 2001	Retrospective cohort (Denmark)	Inpatient; population-based	1970-1996 (11.7)	2337	92.0% girls and women; most aged 10-24 y; NA; NA	27	All incidence	Yes	*ICD-8* code 306.5	*ICD-7* codes 104-205	General population	SIR	Age, sex, and calendar year
Korndörfer et al,^30^ 2003	Retrospective cohort (United States)	Community; population-based	1935-2000 (27.1)	208	92.8% women; mean age, 21.5 y; 100% white; NA	3 Cancer-related deaths	All mortality	No	*DSM-III-R* codes unreported	*ICD-9* codes	General population	SMR	Age and sex
Michels and Ekbom,^13^ 2004	Retrospective cohort (Sweden)	Inpatient; population-based	1965-2000 (13.3)	7303	100% girls and women; most aged <20 y; NA; 73% nulliparous	52	All incidence	Yes	*ICD-7* codes 316.99 and 784.09; *ICD-8* code 306.5; *ICD-9* code 307B	*ICD-7* codes	General population	SIR	Age and calendar year
Karaminis et al,^23,24,25^ 2014	Retrospective cohort (Sweden)	Inpatient; population-based	1973-2003 (15.2)	6009	100% women; mean age, 26.4 y; NA; 65% nulliparous	74	All incidence and all mortality	Yes	*ICD-8* code 306.5; *ICD-9* code 307B; *ICD-10* codes F50.0 and F50.1	*ICD-7* codes 140-207; *ICD-8* codes 140-209; *ICD-9* codes: 140-208; *ICD-10* codes: C00-C96	General population	SIR and SMR	Age and calendar year
Brewster et al,^31^ 2015	Retrospective cohort (United Kingdom)	Inpatient; population-based	1981-2012 (13.9)	2138	60.5% women; mean age, 23.8 y; NA; NA	15	Specific incidence	Yes	*ICD-9* codes 307.1 and 307.5; *ICD-10* code F50	*ICD-9* codes 150, 151, 162; *ICD-10* codes C15, C16, C33-C34	General population	SIR	Age, sex, calendar year, and socioeconomic status
Mellemkjaer et al,^29,32^ 2015	Retrospective cohort (Denmark, Finland and Sweden)	Inpatient; population-based	1968-2011 (12.6)	24 332	93.1% women; 63% <20 y; NA; 64% nulliparous	389	All incidence	Yes	*ICD-8* code 306.5; *ICD-9* code 307; *ICD-10* codes F50.0 and F50.1	*ICD-9* codes; *ICD-10* codes	General population	IRR and HR	Age, sex, calendar year, and country
O’Brien et al,^33^ 2017	Prospective cohort (United States)	Community; multicenter	2003-2009 (5.4)	275	100% women; mean age, 51.7 y; 90% white, 4% African American, 4% Hispanic, 4% other; NA	2	Specific incidence	Yes	Interview with patient report, *DSM-5*	Validated diagnoses using medical records	Participants without history of eating disorder	HR	Age, education, and race/ethnicity

Abbreviations: AN, anorexia nervosa; *DSM-III-R*, *Diagnostic and Statistical Manual of Mental Disorders *(Third Edition Revised); *DSM-5*, *Diagnostic and Statistical Manual of Mental Disorders* (Fifth Edition); HR, hazard ratio; *ICD*, *International Classification of Diseases*; IRR, incidence rate ratio; NA, not available; SIR, standardized incidence ratio; SMR, standardized mortality ratio.

**Figure 2.**
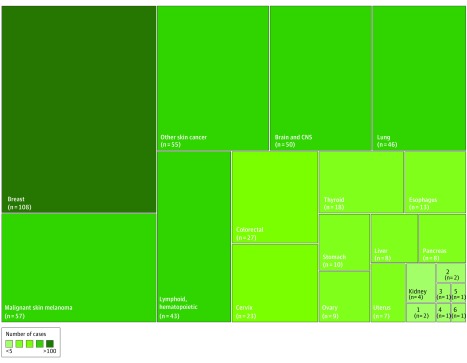
Treemap Summarizing the Amount of Data According to Site-Specific Cancers 1 indicates gallbladder; 2, lip and oral cavity; 3, bladder; 4, bone; 5, prostate; 6, testicular; and CNS, central nervous system.

There were 79 individual study estimates for all types of cancer and 22 site-specific cancer outcomes (eTable 5 in the Supplement). All 7 studies provided adjusted risk estimates (eg, adjusted for age, sex, and calendar year). Breast cancer was the most studied site-specific outcome, accounting for 108 cases (19.8%) in 5 studies. Bladder, bone, prostate, and testicular cancer only accounted 1 case each, representing 0.7% of all cancer cases (Figure 2). Regarding the methodological quality (eTable 6 in the Supplement), 6 articles (86%) had low risk of bias and 1 had moderate risk of bias (Newcastle-Ottawa Scale values, 5-8).

### Meta-analyses

**Figure 3.**
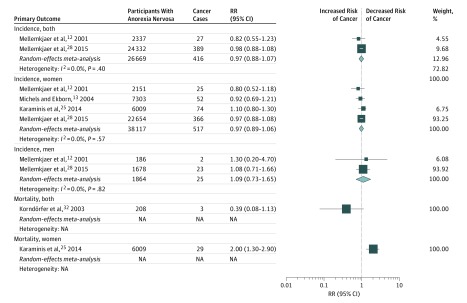
Association of Anorexia Nervosa With Risk of Cancer The size of each box indicates the effect of each study by weight assigned using the random-effects model; diamond, estimated effect size; width of diamond, the precision of the estimate (95% CI); NA, not applicable; and RR, risk ratio.

We found no difference in all cancer incidence between patients with anorexia nervosa and the general population (4 studies in women; RR, 0.97; 95% CI, 0.89-1.06; *P* = .53; *I*^2^, 0%; 95% PI, 0.80-1.18; moderate confidence) (Table 2 and Figure 3). Only 2 studies included data on cancer risk for male participants (RR, 1.09; 95% CI, 0.73-1.65; *P* = .67; *I*^2^, 0%; low confidence), and 2 included data on cancer risk among both sexes combined (RR, 0.97; 95% CI, 0.88-1.07; *P* = .54; *I*^2^, 0%; low confidence). Meta-analysis for all cancer mortality was not possible, with only 1 study in women (RR, 2.00; 95% CI, 1.30-2.90; *P* = .001; low confidence) and 1 study in both sexes combined (RR, 0.39; 95% CI, 0.08-1.13; *P* = .16; low confidence).

**Table 2.** Main Results of Meta-analysis for Association of Anorexia Nervosa With Cancer^a^

Outcome of Interest	Studies, No.	Participants With Anorexia Nervosa, No.	Cancer Cases, No.	Pooled RR (95% CI)	RR of Largest Study (95% CI)	*P* Value for Effect Estimate	*I*^2^ (95% CI)	95% Prediction Interval^b^	*P* Value for Heterogeneity	Confidence
**All Cancer**										
Incidence in both sexes	2	26 669	416	0.97 (0.88-1.07)	0.98 (0.88-1.08)	.54	0 (NA)	NA	.40	Low (risk unlikely)
Incidence in women	4	38 117	517	0.97 (0.89-1.06)	0.97 (0.88-1.08)	.53	0 (0-85)	0.80-1.18	.57	Moderate (risk unlikely)
Incidence in men	2	1864	25	1.09 (0.73-1.65)	1.08 (0.71-1.66)	.67	0 (NA)	NA	.82	Low (not conclusive)
**Site-Specific Cancer**										
Brain and central nervous system										
Incidence in both sexes	2	26 669	45	2.31 (0.58-9.12)	1.34 (0.95-1.89)	.23	72 (NA)	NA	.06	Low (not conclusive)
Incidence in women	3	30 814	43	1.14 (0.81-1.62)	1.20 (0.80-1.70)	.45	0 (0-90)	0.12-10.89	.78	Low (not conclusive)
Incidence in men	2	1864	9	4.52 (0.89-22.97)	2.30 (1.00-5.20)	.07	68 (NA)	NA	.08	Low (not conclusive)
Breast										
Incidence in women	5	38 392	108	0.60 (0.50-0.74)	0.60 (0.50-0.80)	<.001	0 (0-79)	0.44-0.83	.66	High (convincing)
Mortality in women	2	28 663	18	1.22 (0.31-4.77)	2.10 (1.30-3.60)	.78	69 (NA)	NA	.07	Low (not conclusive)
Cervix, incidence in women	2	24 805	23	0.69 (0.45-1.07)	0.70 (0.40-1.00)	.10	0 (NA)	NA	.85	Low (not conclusive)
Esophagus, incidence in women	2	24 805	6	6.10 (2.30-16.18)	5.10 (1.80-14.60)	<.001	0 (NA)	NA	.35	Low (not conclusive)
Lip and oral cavity, incidence in women	2	8160	2	2.00 (0.27-14.73)	3.20 (0.10-17.40)	.49	0 (NA)	NA	.57	Low (not conclusive)
Lung										
Incidence in both sexes	2	26 650	37	1.50 (1.06-2.12)	1.57 (1.07-2.30)	.02	0 (NA)	NA	.58	Low (suggestive)
Incidence in women	3	30 814	38	1.77 (1.25-2.50)	1.60 (1.10-2.40)	.001	0 (0-90)	0.19-16.46	.54	Low (suggestive)
Lymphoid and hematopoietic, incidence in women	3	30 814	36	1.83 (0.78-4.30)	1.10 (0.80-1.60)	.16	57 (0-88)	0-17025	.10	Low (not conclusive)
Malignant skin melanoma, incidence in women	3	30 814	56	1.10 (0.84-1.44)	1.10 (0.80-1.50)	.49	0 (0-90)	0.19-6.39	.38	Low (not conclusive)
Other skin cancer, incidence in women	2	28 663	50	1.12 (0.82-1.52)	1.10 (0.80-1.50)	.48	0 (NA)	NA	.56	Low (not conclusive)
Pancreas, incidence in women	2	24 805	7	1.94 (0.87-4.34)	1.80 (0.80-4.30)	.10	0 (NA)	NA	.54	Low (not conclusive)
Stomach, incidence in women	2	8160	9	1.51 (0.73-3.11)	1.40 (0.60-2.70)	.27	0 (NA)	NA	.48	Low (not conclusive)
Thyroid gland, incidence in women	3	30 814	18	0.97 (0.59-1.60)	0.90 (0.50-1.70)	.91	0 (0-90)	0.04-24.85	.88	Low (not conclusive)

Abbreviations: NA, not available; RR, risk rate.

^a^ Cancer outcomes with 2 or more studies for meta-analysis.

^b^ Prediction intervals and *I*^2^ 95% CIs were inestimable with fewer than 3 studies.

In terms of the 22 site-specific cancers, a minority of associations of anorexia nervosa with risk of developing cancer or death of cancer have supporting evidence (Table 2; eTable 8 in the Supplement). Anorexia nervosa was associated with decreased breast cancer incidence compared with the general female population (5 studies in women; RR, 0.60; 95% CI, 0.50-0.80; *P* < .001; *I*^2^, 0%; 95% PI, 0.44-0.83; high confidence). Conversely, we found anorexia nervosa was potentially associated with an increased risk of developing lung cancer (3 studies in women; RR, 1.50; 95% CI, 1.06-2.12; *P* = .001; *I*^2^, 0%; 95% PI, 0.19-16.46; low confidence) and esophageal cancer (2 studies in women; RR, 6.10; 95% CI, 2.30-16.18; *P* < .001; *I*^2^, 0%; low confidence).

No differences were found for any of the other specific sites of cancer investigated through meta-analyses, including cancer of the brain and central nervous system, cervical cancer, lip and oral cavity cancer, lymphoid and hematopoietic cancer, malignant skin melanoma, other skin cancer, pancreatic cancer, stomach cancer, and cancer of the thyroid gland (Table 2). Meta-analysis for 10 site-specific cancers was not possible; only 1 study included outcome data on bladder cancer, bone and articular cartilage cancer, colorectal cancer, gallbladder cancer, kidney cancer, liver cancer, ovarian cancer, prostate cancer, testicular cancer, and uterus cancer (eTable 5 in the Supplement).

### Additional Analyses

The full details of the additional analyses are reported in eFigures 1-9 in the Supplement. We noted that the results were potentially different when we examined the outcomes by considering cancer types according to association with smoking or sex hormones. An increased risk of smoking-related cancer incidence was observed in women with anorexia nervosa (4 studies; RR, 1.59; 95% CI, 1.17-2.16; *P* = .003; *I*^2^, 58%) but not with non–smoking-related cancers (4 studies; RR, 0.89; 95% CI, 0.73-1.09; *P* = .27; *I*^2^, 40%). In addition, anorexia nervosa was associated with decreased risk of developing cancers occurring in hormone-sensitive tissues compared with those without anorexia nervosa or the general population (5 studies; RR, 0.69; 95% CI, 0.55-0.87; *P* = .002; *I*^2^, 27%).

For the secondary outcome of breast cancer incidence, findings remained largely consistent after considering parity status (3 studies in parous women; RR, 0.48; 95% CI, 0.36-0.62; *P* < .001; *I*^2^, 0%; 3 studies in nulliparous women: RR, 0.74; 95% CI, 0.55-0.99; *P* = .05; *I*^2^, 0%) and age at receiving first diagnosis of anorexia nervosa (3 studies in women aged <20 years: RR, 0.30; 95% CI, 0.08-1.13; *P* = .08; *I*^2^, 76%; 3 studies in women aged ≥20 years; RR, 0.63; 95% CI, 0.47-0.84; *P* = .002; *I*^2^, 0%), but some analyses may have led to insufficient statistical power.

No differences in conclusions were observed in post hoc sensitivity analyses after excluding studies that had potential overlapping populations. Finally, given the number of individual study estimates available per outcome (<10 studies) and reporting variability for specific sites of cancer events, assessment of small study effects (publication bias) for pooled analyses was considered not feasible.^15^

## Discussion

To our knowledge, this is the first systematic review and meta-analysis to assess the totality of evidence regarding the association of anorexia nervosa with cancer risk. Although the scientific understanding of the extent of the epidemiological evidence underlying the associations of anorexia nervosa with cancer has developed within the past decades, only a minority of associations of anorexia nervosa with risk of developing cancer in specific cancer sites have supporting or suggestive evidence, to our knowledge. Fewer studies exist for the association of anorexia nervosa with risk of death from cancer, to our knowledge.

Our analysis does not support the possibility of a true inverse association of anorexia nervosa with cancer in general. Anorexia nervosa was associated with some cancers but not others. This may illustrate the fact that cancer is a complex, multifaceted, and heterogeneous disease with considerable variations in the scale of incidence (and mortality) of cancer types worldwide,^1,5,6^ particularly among young people.^34^ Breast cancer is the major contributor to the cancer burden among young adults globally.^5,34^ Findings from our meta-analysis suggest that anorexia nervosa was associated with decreased breast cancer incidence compared with the general female population, with high confidence. Conversely, we found that anorexia nervosa was potentially associated with increased risk of developing lung and esophageal cancer, but the evidence was judged as only low confidence.

The mechanisms of these patterns of risk have yet to be fully characterized. Most cancers are the result of many risk factors. Anorexia nervosa is a disorder associated with alterations in hormonal profiles.^2,3,35^ Reduced serum concentrations of estradiol and insulin-like growth factor 1 together with decreased lifetime exposure to estrogens owing to delayed puberty and hastened menopause might partially explain why young women with anorexia nervosa may have reduced risk of breast cancer and other hormone-sensitive tissue cancers. The extreme caloric restriction in anorexia nervosa^10,11,12,13,14,36^ could also affect the risk of some specific cancers but not cancer incidence in general. We noted inverse associations of breast cancer incidence with anorexia nervosa even after considering age at receiving first diagnosis of anorexia nervosa and parity status. Parity is widely recognized as protective against breast cancer, but breast cancer risk may be increased shortly after childbirth. A 2019 meta-analysis^37^ showed that compared with nulliparous women, parous women had an increased risk of breast cancer that peaked about 5 years after giving birth before decreasing after longer than 30 years. The overall pattern was driven by estrogen receptor–positive breast cancers.^37^

Anorexia nervosa is a marker of calorie restriction characterized by an abnormally low body weight or low BMI. Various forms of reduced caloric intake, such as calorie restriction or fasting, have demonstrated a range of potential beneficial effects to help prevent malignancies and increase the efficacy of cancer therapies.^10,11,36,38^ Previous studies have shown associations of high BMI with many cancers.^7,8,9^ However, the decreased cancer risk we observed in our study was limited to estrogen-responsive tumors, and this may suggest that the caloric restriction seen in anorexia nervosa does not confer substantial cancer protection. The biological mechanisms underlying the association of BMI with the risk of breast cancer are complex, with opposing effects on premenopausal and postmenopausal risk of breast cancer.^7,8,9,39,40,41^ A 2016 International Agency for Research on Cancer report^42^ acknowledged consistent inverse associations of BMI with the risk of premenopausal breast cancer but inconsistent findings from studies that evaluated the effect of waist circumference or weight gain.

An increased risk was found for smoking-related cancers in women. However, the increased risk of developing lung or esophageal cancer does not seem to be attributable to a higher prevalence of smoking among women with anorexia nervosa. For example, in a 2016 meta-analysis^43^ evaluating the association of smoking prevalence with eating disorders, people with bulimia nervosa were significantly more likely to be lifetime smokers than healthy controls but not people with anorexia nervosa. Similarly, there was no difference in the proportion of current smokers among people with anorexia nervosa and healthy controls, while there was an increased proportion of current smokers among people with bulimia nervosa compared with healthy controls. These findings suggest that there may be a need for continued surveillance of symptoms suggestive of cancers of the respiratory tract system among patients with anorexia nervosa and for identification and evaluation of evidence-based health promotion interventions in patients with anorexia nervosa.

A number of noncausal explanations have been offered to account for the association of anorexia nervosa with cancer. Psychiatric comorbidity is common in anorexia nervosa^2,4^ and could affect cancer development or diagnosis. The presence of dense breast tissue may increase the risk of developing breast cancer.^44,45^ However, the role that the density of breast tissue may play in cancer development remains underexplored in anorexia nervosa and requires further research. Patients with anorexia nervosa might experience decreased rates of cancer screening as their condition worsens. Symptoms of anorexia nervosa might mask symptoms of cancer, and patients might be less willing to undergo examinations, such as mammograms. Some may suggest that since patients with anorexia nervosa have a higher mortality rate than the general population,^23,24,46,47^ those who survive anorexia nervosa may also be less susceptible to dying of cancer compared with the general population. However, we found no decreased all-cancer incidence rates in cohort studies, so this is an unlikely explanation.

### Limitations

There are some limitations to be noted regarding our study. Similar to other meta-analyses of observational studies, there is methodological and clinical heterogeneity in the included studies in design, patient populations, and outcomes. We used study-level data instead of individual patient data, so the small number of studies and events for most site-specific cancers limited the sensitivity analyses that could be conducted to account for heterogeneity in the absence of patient-level data. Furthermore, our results might have limited generalizability because the cohort studies were mostly conducted in Western and Northern countries, and there were no reports from low- to middle-income countries with lower prevalence and incidence of anorexia nervosa. Meta-analysis of observational studies are prone to bias and confounding factors, including family history of cancer, smoking, alcohol, physical activity, and nutritional deficiency, that are inherent in the individual studies. Although we adopted reproducible definitions that will enhance cross-study comparisons, our study was challenged by the reporting of events in studies. None of the individual studies examined and reported all of the cancer outcomes of interest. Also, incomplete reporting was common in the literature and limited our possibilities to optimally synthesize the data for some specific cancers. It is important that future observational studies thoroughly report the methods and results for all outcomes of interest to ensure inclusion in future evidence syntheses. Reporting guidelines, such as the Strengthening the Reporting of Observational Studies in Epidemiology (STROBE) reporting guideline,^48^ should be rigorously adopted and implemented for individual study reports. Small study effects (ie, publication bias) were not quantitatively assessed, as there were inadequate numbers of included studies to properly assess a funnel plot or use more sophisticated regression-based methods. Further insights may be gained from analyses of large clinical and administrative databases with the power to examine the association of anorexia nervosa with specific cancers.

## Conclusions

The association of anorexia nervosa with the risk of cancer has been studied in the biomedical literature. In general, we had low confidence in observational associations of anorexia nervosa with the risk of 20 outcomes, but there was supporting evidence from meta-analysis for 2 outcomes (all cancer combined and breast cancer). Other associations could also be valid (eg, lung cancer and esophageal cancer), but there is still uncertainty about the associations and the role of modifiable risk factors. There is still paucity of epidemiological data in many of the explored associations, and the number of individual studies is limited for most cancer outcomes. To draw firmer conclusions, we need more prospective cohort studies and large collaborations with better assessment of the complex connections among anorexia nervosa, losing body weight, and cancer.

## Notes

Supplement. **eTable 1.** Definitions of Specific Cancer Site Outcomes **eTable 2.** Methods Clarifications and Modifications From the Protocol **eTable 3.** Search Strategy **eTable 4.** List of Excluded Studies **eTable 5.** Results of Individual Studies **eTable 6.** Methodological Quality Assessment of Included Studies Using Newcastle-Ottawa Scale **eTable 7.** Grading Certainty or Credibility of Evidence for Summary Estimates **eTable 8.** Summary Statistics of Risk of Cancer for People With Anorexia Nervosa **eFigure 1.** Meta-analysis of Breast Cancer Incidence and Mortality **eFigure 2.** Meta-analysis of Breast Cancer Incidence and Subgroup Analysis by Parity Status and Age at First Diagnosis of Anorexia Nervosa **eFigure 3.** Meta-analysis of Lung Cancer Incidence **eFigure 4.** Meta-analysis of Lymphoid and Hematopoietic Cancer Incidence and Mortality **eFigure 5.** Meta-analysis of Malignant Skin Melanoma Incidence and Mortality **eFigure 6.** Meta-analysis of Smoking-Related Cancers **eFigure 7.** Meta-analysis of Non–Smoking-Related Cancers **eFigure 8.** Meta-analysis of Cancers Occurring in Hormone-Sensitive Tissues **eFigure 9.** Sensitivity Analysis
