# Supplementary material for: Association of Anorexia Nervosa With Risk of Cancer: A Systematic Review and Meta-analysis
Source: JAMA Netw Open. 2019 Jun 7;2(6):e195313. doi: 10.1001/jamanetworkopen.2019.5313 (PMC6563572; doi:10.1001/jamanetworkopen.2019.5313)
Supplement: Supplement. — eTable 1. Definitions of Specific Cancer Site Outcomes eTable 2. Methods Clarifications and Modifications From the Protocol eTable 3. Search Strategy eTable 4. List of Excluded Studies eTable 5. Results of Individual Studies eTable 6. Methodological Quality Assessment of Included Studies Using Newcastle-Ottawa Scale eTable 7. Grading Certainty or Credibility of Evidence for Summary Estimates eTable 8. Summary Statistics of Risk of Cancer for People With Anorexia Nervosa eFigure 1. Meta-analysis of Breast Cancer Incidence and Mortality eFigure 2. Meta-analysis of Breast Cancer Incidence and Subgroup Analysis by Parity Status and Age at First Diagnosis of Anorexia Nervosa eFigure 3. Meta-analysis of Lung Cancer Incidence eFigure 4. Meta-analysis of Lymphoid and Hematopoietic Cancer Incidence and Mortality eFigure 5. Meta-analysis of Malignant Skin Melanoma Incidence and Mortality eFigure 6. Meta-analysis of Smoking-Related Cancers eFigure 7. Meta-analysis of Non–Smoking-Related Cancers eFigure 8. Meta-analysis of Cancers Occurring in Hormone-Sensitive Tissues eFigure 9. Sensitivity Analysis [file jamanetwopen-2-e195313-s001.pdf]

Supplementary Online Content

Catalá-López F, Forés-Martos J, Driver JA, et al. Association of anorexia nervosa with risk of cancer: a systematic review and meta-analysis. *JAMA Netw Open*. 2019;2(6):195313. doi:10.1001/jamanetworkopen.2019.5313

eTable 1. Definitions of Site-Specific Cancer Outcomes

eTable 2. Methods Clarifications and Modifications From the Protocol

eTable 3. Search Strategy

eTable 4. List of Excluded Studies

eTable 5. Results of Individual Studies

eTable 6. Methodological Quality Assessment of Included Studies Using Newcastle-Ottawa Scale

eTable 7. Grading Certainty or Credibility of Evidence for Summary Estimates

eTable 8. Summary Statistics of Risk of Cancer for People With Anorexia Nervosa

eFigure 1. Meta-analysis of Breast Cancer Incidence and Mortality

eFigure 2. Meta-analysis of Breast Cancer Incidence and Subgroup Analysis by Parity Status and Age at First Diagnosis of Anorexia Nervosa

eFigure 3. Meta-analysis of Lung Cancer Incidence

eFigure 4. Meta-analysis of Lymphoid and Hematopoietic Cancer Incidence and Mortality

eFigure 5. Meta-analysis of Malignant Skin Melanoma Incidence and Mortality

eFigure 6. Meta-analysis of Smoking-Related Cancers

eFigure 7. Meta-analysis of Non–Smoking-Related Cancers

eFigure 8. Meta-analysis of Cancers Occurring in Hormone-Sensitive Tissues

eFigure 9. Sensitivity Analysis

This supplementary material has been provided by the authors to give readers additional information about their work.

© 2019 Catalá-López F et al. *JAMA Network Open.*

eTable 1. Definitions of Site-Specific Cancer Outcomes

Specific cancer-site	ICD-9 code	ICD-10 code
Esophageal cancer†	150-150.9	C15-C15.9
Stomach cancer†	151-151.9, 209.23	C16-C16.9
Liver cancer†	155-155.3	C22-C22.9
Larynx cancer†	161-161.9, 162.1	C32-C32.9
Tracheal, bronchus and lung cancers†	162, 162.0, 162.2-162.9, 209.21	C33-C34.9
Breast cancer	174-175.9	C50-C50.929
Cervical cancer†	180-180.9	C53-C53.9
Uterine cancer	182-182.8	C54-C54.9
Prostate cancer	185-185.9	C61-C61.9
Colon and rectum cancer†	153-154.9, 155.5, 155.9, 209.1-209.17	C18-C20.0, C20.9-C21.8
Lip and oral cavity cancer†	140-145.9	C00-C08.9
Nasopharynx cancer†	147-147.9	C11-C11.9
Other pharynx cancer†	146-146.9, 148-148.9	C09-C10.9, C12-C13.9
Gallbladder and biliary tract cancer	156-156.9, 209.25-209.27	C20.8, C23-C24.9
Pancreatic cancer†	157-157.9	C25-C25.9
Malignant skin melanoma	172-172.9	C43-C43.9, C4A
Ovarian cancer†	183, 183.0	C56-C56.9
Testicular cancer	186-186.9	C62-C62.92
Kidney cancer†	189.0, 189.1, 209.24	C64-C65.9
Bladder cancer†	188-188.9	C67-C67.9
Brain and nervous system cancer	191-192.9	C70-C72.9
Thyroid cancer	193-192.9	C73-C73.9
Mesothelioma	58-158.9, 163-163.3, 163.8, 163.9	C45-C45.9
Hodgkin lymphoma	201-201.98	C81-C81.99
Non-Hodgkin lymphoma	200-200.9, 202-202.98	C82-C86.6, C96-C97.9
Multiple myeloma	203-203.9	C88-C90.32
Leukaemia†	204-208.92	C91-C95.92

© 2019 Catalá-López F et al. *JAMA Network Open.*

Other neoplams	152-152.9, 160-160.9,164-164.9, 170-171.9, 181-181.9, 182.9, 183.2-183.8, 184.0-184.4, 184.8, 187.1-187.8, 189.2-189.8, 190-190.9, 194-194.8	C17-C17.9, C30-C31.9, C37-C38.8, C40-C41.9, C47-C49.9, C50.12-C50.129, C51-C52.9, C57-C57.8, C58, C58.0, C60-C60.9, C63-C63.8, C66-C66.9, C68.0-C68.8, C69-C69.92, C74-C75.8
Other benign neoplasm	209.4, 209.40, 209.41, 209.42, 209.43, 211.2, 211.8, 212.0, 212.4, 212.5, 212.6, 212.7, 212.8, 213, 213.0, 213.1, 213.2, 213.3, 213.4, 213.5, 213.6, 213.7, 213.8, 213.9, 214.2, 214.3, 214.4, 214.8, 214.9, 221.0, 221.1, 221.2, 221.8, 222.1, 222.8, 223.2, 223.8, 223.81, 223.89, 224, 224.0, 224.1, 224.2, 224.3, 224.4, 224.5, 224.6, 224.7, 224.8, 224.9, 227, 227.0, 227.1, 227.3, 227.4, 227.5, 227.6, 227.8, 227.9, 228, 228.0, 228.00, 228.01, 228.02, 228.03, 228.04, 228.09, 228.1, 228.9, 229.0, 229.8, 230.7, 230.8, 233.31, 233.32, 233.4, 233.5, 234.0, 234.5, 234.8, 235.4, 235.8, 236.1, 236.99, 238.0, 238.1, 238.4, 238.5, 238.6, 238.7, 238.71, 238.72, 238.73, 238.74, 238.75, 238.76, 238.77, 238.79, 238.8, 239.2, 623.0, 623.1, 623.7, 210.8	D07.1, D07.2, D07.4, D07.5, D09.2, D09.20, D09.21, D09.22, D10.7, D13.2, D13.3, D13.30, D13.39, D14.0, D15, D15.0, D15.1, D15.2, D15.7, D15.9, D16, D16.0, D16.00, D16.01, D16.02, D16.1, D16.10, D16.11, D16.12, D16.2, D16.20, D16.21, D16.22, D16.3, D16.30, D16.31, D16.32, D16.4, D16.5, D16.6, D16.7, D16.8, D16.9, D17.9, D18, D18.0, D18.00, D18.01, D18.02, D18.03, D18.09, D18.1, D19, D19.0, D19.1, D19.7, D19.9, D20, D20.0, D20.1, D20.9, D21, D21.0, D21.1, D21.10, D21.11, D21.12, D21.2, D21.20, D21.21, D21.22, D21.3, D21.4, D21.5, D21.6, D21.9, D28.0, D28.1, D28.2, D28.7, D29.0, D30.2, D30.20, D30.21, D30.22, D30.4, D30.7, D30.8, D31, D31.0, D31.00, D31.01, D31.02, D31.1, D31.10, D31.11, D31.12, D31.2, D31.20, D31.21, D31.22, D31.3, D31.30, D31.31, D31.32, D31.4, D31.40, D31.41, D31.42, D31.5, D31.50, D31.51, D31.52, D31.6, D31.60, D31.61, D31.62, D31.9, D31.90, D31.91, D31.92, D35, D35.0, D35.00, D35.01, D35.02, D35.1, D35.2, D35.3, D35.4, D35.5, D35.6, D35.7, D35.8, D35.9, D36, D36.1, D36.10, D36.11, D36.12, D36.13, D36.14, D36.15, D36.16, D36.17, D36.7, D37.2, D38.2, D38.3, D38.4, D38.5, D39.2, D39.7, D39.8, D41.2, D41.20, D41.21, D41.22, D41.3, D44.1, D44.10, D44.11, D44.12,

© 2019 Catalá-López F et al. *JAMA Network Open.*

		D44.2, D44.3, D44.4,D44.5,D44.6,D44.7,D44.8,D45, D45.0, D45.9, D46, D46.0, D46.1, D46.2, D46.20, D46.21, D46.22, D46.3, D46.4, D46.5, D46.7, D46.9, D47, D47.3, D47.4, D47.5, D47.7, D48.0, D48.1, D48.2, D48.3, D48.4, D48.7, D49.81

Note: List of International Classification of Diseases (ICD) codes mapped to the Global Burden of Disease cause list for cancer.

†Smoking-related cancer sites: lip and oral cavity cancer, esophageal cancer, stomach cancer, liver cancer, pancreatic cancer, nasopharynx cancer, larynx cancer, lung cancer (tracheal, bronchus and lung cancers), ovarian cancer, cervical cancer, colorectal cancer, kidney cancer, bladder cancer, leukaemia.

"Lymphoid, hematopoietic and related tissue" cancer (ICD-10 codes = C81-C96; ICD-9 codes = 200-208) were reported in 2 studies.

© 2019 Catalá-López F et al. *JAMA Network Open.*

eTable 2. Methods Clarifications and Modifications From the Protocol

The protocol for this meta-analysis has been registered in PROSPERO (No. CRD42017067462), and published in the open-access journal *Systematic Reviews*, available at: https://systematicreviewsjournal.biomedcentral.com/articles/10.1186/s13643-017-0540-5
**Clarification 1: Additional analyses**<br>Page 4 of the published protocol: "*We will conduct not only subgroup analyses for cancer types according to relationship with smoking (smoking-related cancer sites or other cancer sites) (see Additional file 3) but also meta-regression analyses considering the percentage of smokers (e.g. past or current smoker)*."<br><br>Clarification: We originally planned to conduct meta-regression analyses considering smoking as a study-level covariate. However, during the data extraction process we observed that data on smoking story were incompletely and inconsistently reported among epidemiological studies. Considering the number of studies with missing information, we therefore decided not to apply this approach. This decision was made during the data extraction and before the data analysis.<br><br>The final list of smoking-related cancer sites considered in the subgroup analyses has been updated by the review team replacing "uterine cancer" by "colorectal cancer" (based on most updated information from WHO/IARC[1] and Cancer UK Research[2]). The final list of smoking-related cancer sites follows: lip and oral cavity cancer, oesophageal cancer, stomach cancer, liver cancer, pancreatic cancer, nasopharynx cancer, larynx cancer, lung cancer (tracheal, bronchus and lung cancers), ovarian cancer, cervical cancer, colorectal cancer, kidney cancer, bladder cancer, and leukaemia.<br><br>[1]https://cancer-code-europe.iarc.fr/<br>[2]http://www.cancerresearchuk.org/about-cancer/causes-of-cancer/smoking-and-cancer
**Clarification 2: Additional analyses**<br>Page 4 of the published protocol: "*We plan to conduct subgroup analyses by (…) study design (cohort or case–control; prospective, or retrospective), follow-up (0–1, >1–5, or >5 years), setting (mixed, inpatient, outpatient, or community), ethnicity (e.g., Asian or non-Asian), population-based (yes or no), country economic status (developed or developing countries according to International Monetary Fund), year of publication (before 2000 or in 2000 and after), study quality (high or low-moderate risk of bias), adjustment for confounding variables (age, sex or other), and sample size (<500, 500–1000 or >1000 participants)*."<br><br>Clarification: Pre-planned subgroup analyses (such as "study design", "setting", "ethnicity", "population-based (Y/N)", "country economic status", "year of publication", "study quality", "adjustment for confounders", "sample size") could not be carried out for pooled analyses as there were not enough data for establishing well-balanced subgroups.<br><br>Page 5 of the published protocol: "*If sufficient studies are identified, we will perform cumulative meta-analysis in the order of publication year* (…)".<br><br>Clarification: Given the number of individual study estimates available per outcome (less than 10 studies), cumulative meta-analysis was considered not feasible.
**Clarification 3: Additional analysis**<br>Page 5 of the published protocol: "*Small study effects will be assessed by inspection of the funnel plots for asymmetry and with Egger's test and Begg's test, with the results considered to indicate potential small study effects when* P *< 0.10*."<br><br>Clarification: Given the number of individual study estimates available per outcome (less than 10 studies), small study effects/publication bias assessment (e.g. funnel plots, Begg and Egger test) was considered not feasible. See Cochrane recommendations here: https://handbook-5-

© 2019 Catalá-López F et al. *JAMA Network Open.*

1.cochrane.org/chapter_10/10_4_3_1_recommendations_on_testing_for_funnel_plot_asymmetry.htm

eTable 3. Search Strategy

eTable 3a. Key terms for **PubMed/MEDLINE** search.

Search	Query	Items
#1	anorexia nervosa OR eating disorder*	35167
#2	cancer* OR carcinoma* OR neoplasia* OR tumor* OR neoplasm* OR maligna*	3963959
#3	epidemiolog* OR cohort stud* OR longitudinal stud* OR case-control stud*	2391184
#4	#1 AND #2 AND #3<br>No limits	**160**

eTable 3b. Key terms for **SCOPUS** search.

Search	Query	Items
#1	(TITLE-ABS-KEY ("Anorexia nervosa" OR "eating disorder*")) AND (TITLE-ABS-KEY (cancer* OR carcinoma* OR neoplasia* OR tumor* OR neoplasm* OR maligna*)) AND (TITLE-ABS-KEY (epidemiolog* OR cohort* OR "longitudinal stud*" OR "case-control"))	**211**

eTable 3c. Key terms for **Embase** search.

Search	Query	Items
#1	'anorexia nervosa':ti,ab,kw OR 'eating disorder':ti,ab,kw AND ('maligna*':ti,ab,kw OR 'neoplasm*':ti,ab,kw OR 'neoplasia*':ti,ab,kw OR 'tumor*':ti,ab,kw OR 'cancer*':ti,ab,kw OR 'carcinoma*':ti,ab,kw) AND ('epidemiolog*':ti,ab,kw OR 'cohort*':ti,ab,kw OR 'longitudinal stud*':ti,ab,kw OR 'case-control':ti,ab,kw)	**38**

eTable 3d. Key terms for **Web of Science** Core collection search.

Search	Query	Items
#1	TS=("Anorexia nervosa" OR "eating disorder*") AND TS=(cancer* OR carcinoma* OR neoplasia* OR tumor* OR neoplasm* OR maligna*) AND TS=(epidemiolog* OR cohort* OR "longitudinal stud*" OR "case-control")<br>Índices=SCI-EXPANDED, SSCI, A&HCI, CPCI-S, CPCI-SSH, BKCI-S, BKCI-SSH, ESCI, CCR-EXPANDED, IC Período de tiempo=Todos los años	**85**

© 2019 Catalá-López F et al. *JAMA Network Open.*

eTable 4. List of Excluded Studies

Reference	Cause
1. Tolstrup K, Brinch M, Isager T, Nielsen S, Nystrup J, Severin B, Olesen NS. Long-term outcome of 151 cases of anorexia nervosa. The Copenhagen Anorexia Nervosa Follow-Up Study. Acta Psychiatr Scand. 1985 Apr;71(4):380-7. PubMed PMID: 4003103.	Cancer not outcome
2. Patton GC. Mortality in eating disorders. Psychol Med. 1988 Nov;18(4):947-51. PubMed PMID: 3270837.	Cancer not outcome
3. Lucas AR, Beard CM, O'Fallon WM, Kurland LT. 50-year trends in the incidence of anorexia nervosa in Rochester, Minn.: a population-based study. Am J Psychiatry. 1991 Jul;148(7):917-22. PubMed PMID: 2053633.	Cancer not outcome
4. Crisp AH, Callender JS, Halek C, Hsu LK. Long-term mortality in anorexia nervosa. A 20-year follow-up of the St George's and Aberdeen cohorts. Br J Psychiatry. 1992 Jul;161:104-7. PubMed PMID: 1638303.	Cancer not outcome
5. Joergensen J. The epidemiology of eating disorders in Fyn County, Denmark, 1977-1986. Acta Psychiatr Scand. 1992 Jan;85(1):30-4. PubMed PMID: 1546545.	Cancer not outcome
6. Møller-Madsen S, Nystrup J. Incidence of anorexia nervosa in Denmark. Acta Psychiatr Scand. 1992 Sep;86(3):197-200. PubMed PMID: 1414412.	Cancer not outcome
7. Norring CE, Sohlberg SS. Outcome, recovery, relapse and mortality across six years in patients with clinical eating disorders. Acta Psychiatr Scand. 1993 Jun;87(6):437-44. PubMed PMID: 8356896.	Cancer not outcome
8. Deter HC, Herzog W. Anorexia nervosa in a long-term perspective: results of the Heidelberg-Mannheim Study. Psychosom Med. 1994 Jan-Feb;56(1):20-7. PubMed PMID: 8197311.	Cancer not outcome
9. Pagsberg AK, Wang AR. Epidemiology of anorexia nervosa and bulimia nervosa in Bornholm County, Denmark, 1970-1989. Acta Psychiatr Scand. 1994 Oct;90(4):259-65. PubMed PMID: 7831995.	Cancer not outcome
10. Chipkevitch E. Brain tumors and anorexia nervosa syndrome. Brain Dev. 1994 May-Jun;16(3):175-9, discussion 180-2. Review. PubMed PMID: 7943600	Study design
11. Eckert ED, Halmi KA, Marchi P, Grove W, Crosby R. Ten-year follow-up of anorexia nervosa: clinical course and outcome. Psychol Med. 1995 Jan;25(1):143-56. PubMed PMID: 7792349.	Cancer not outcome
12. Møller-Madsen S, Nystrup J, Nielsen S. Mortality in anorexia nervosa in Denmark during the period 1970-1987. Acta Psychiatr Scand. 1996 Dec;94(6):454-9. PubMed PMID: 9020999.	Data not abstractabl e
13. Strober M, Freeman R, Morrell W. The long-term course of severe anorexia nervosa in adolescents: survival analysis of recovery, relapse, and outcome predictors over 10-15 years in a prospective study. Int J Eat Disord. 1997 Dec;22(4):339-60. PubMed PMID: 9356884.	Cancer not outcome
14. Crow S, Praus B, Thuras P. Mortality from eating disorders--a 5- to 10-year record linkage study. Int J Eat Disord. 1999 Jul;26(1):97-101. PubMed PMID: 10349590.	Cancer not outcome
15. Emborg C. Mortality and causes of death in eating disorders in Denmark 1970-1993: a case register study. Int J Eat Disord. 1999 Apr;25(3):243-51. PubMed PMID: 10191988.	Cancer not outcome
16. Fichter MM, Quadflieg N, Hedlund S. Twelve-year course and outcome predictors of anorexia nervosa. Int J Eat Disord. 2006 Mar;39(2):87-100. PubMed PMID: 16231345.	Cancer not outcome
17. Herzog DB, Greenwood DN, Dorer DJ, Flores AT, Ekeblad ER, Richards A, Blais MA, Keller MB. Mortality in eating disorders: a descriptive study. Int J Eat Disord. 2000 Jul;28(1):20-6. PubMed PMID: 10800010.	Cancer not outcome
18. Zipfel S, Löwe B, Reas DL, Deter HC, Herzog W. Long-term prognosis in anorexia nervosa: lessons from a 21-year follow-up study. Lancet. 2000 Feb 26;355(9205):721-2. PubMed PMID: 10703806.	Cancer not outcome
19. De Filippo E, Signorini A, Bracale R, Pasanisi F, Contaldo F. Hospital admission and mortality rates in anorexia nervosa: experience from an integrated medical-	Cancer not outcome

© 2019 Catalá-López F et al. *JAMA Network Open.*

	psychiatric outpatient treatment. Eat Weight Disord. 2000 Dec;5(4):211-6. PubMed PMID: 11216129.	
20.	Löwe B, Zipfel S, Buchholz C, Dupont Y, Reas DL, Herzog W. Long-term outcome of anorexia nervosa in a prospective 21-year follow-up study. Psychol Med. 2001 Jul;31(5):881-90. PubMed PMID: 11459385.	Cancer not outcome
21.	Hewitt PL, Coren S, Steel GD. Death from anorexia nervosa: age span and sex differences. Aging Ment Health. 2001 Feb;5(1):41-6. PubMed PMID: 11513012.	Cancer not outcome
22.	Lee S, Chan YY, Hsu LK. The intermediate-term outcome of Chinese patients with anorexia nervosa in Hong Kong. Am J Psychiatry. 2003 May;160(5):967-72. PubMed PMID: 12727702.	Study design
23.	Keel PK, Dorer DJ, Eddy KT, Franko D, Charatan DL, Herzog DB. Predictors of mortality in eating disorders. Arch Gen Psychiatry. 2003 Feb;60(2):179-83. PubMed PMID: 12578435.	Cancer not outcome
24.	Birmingham CL, Su J, Hlynsky JA, Goldner EM, Gao M. The mortality rate from anorexia nervosa. Int J Eat Disord. 2005 Sep;38(2):143-6. PubMed PMID: 16134111.	Study design
25.	Cornali C, Franzoni S, Frisoni GB, Trabucchi M. Anorexia as an independent predictor of mortality. J Am Geriatr Soc. 2005 Feb;53(2):354-5. PubMed PMID: 15673368.	Cancer not outcome
26.	Reas DL, Kjelsås E, Heggestad T, Eriksen L, Nielsen S, Gjertsen F, Götestam KG. Characteristics of anorexia nervosa-related deaths in Norway (1992-2000): data from the National Patient Register and the Causes of Death Register. Int J Eat Disord. 2005 Apr;37(3):181-7. PubMed PMID: 15822079.	Cancer not outcome
27.	Viricel J, Bossu C, Galusca B, Kadem M, Germain N, Nicolau A, Millot L, Vergely N, Lassandre S, Carrot G, Lang F, Estour B. [Retrospective study of anorexia nervosa: reduced mortality and stable recovery rates]. Presse Med. 2005 Nov 19;34(20 Pt 1):1505-10. French. PubMed PMID: 16301961.	Cancer not outcome
28.	Crisp A, Gowers S, Joughin N, McClelland L, Rooney B, Nielsen S, Bowyer C, Halek C, Hartman D, Tattersall M, Hugo P, Robinson D, Atkinson R, Clifton A. Death, survival and recovery in anorexia nervosa: a thirty five year study. Eur Eat Disord Rev. 2006;14(3):168-175.	Data not abstractabl e
29.	Lindblad F, Lindberg L, Hjern A. Improved survival in adolescent patients with anorexia nervosa: a comparison of two Swedish national cohorts of female inpatients. Am J Psychiatry. 2006 Aug;163(8):1433-5. PubMed PMID: 16877658.	Study design
30.	Signorini A, De Filippo E, Panico S, De Caprio C, Pasanisi F, Contaldo F. Long-term mortality in anorexia nervosa: a report after an 8-year follow-up and a review of the most recent literature. Eur J Clin Nutr. 2007 Jan;61(1):119-22. Review. PubMed PMID: 16885933.	Cancer not outcome
31.	Keski-Rahkonen A, Hoek HW, Susser ES, Linna MS, Sihvola E, Raevuori A, Bulik CM, Kaprio J, Rissanen A. Epidemiology and course of anorexia nervosa in the community. Am J Psychiatry. 2007 Aug;164(8):1259-65. PubMed PMID: 17671290.	Cancer not outcome
32.	Wentz E, Gillberg IC, Anckarsäter H, Gillberg C, Råstam M. Adolescent-onset anorexia nervosa: 18-year outcome. Br J Psychiatry. 2009 Feb;194(2):168-74. doi: 10.1192/bjp.bp.107.048686. PubMed PMID: 19182181.	Cancer not outcome
33.	Button EJ, Chadalavada B, Palmer RL. Mortality and predictors of death in a cohort of patients presenting to an eating disorders service. Int J Eat Disord. 2010 Jul;43(5):387-92. doi: 10.1002/eat.20715. PubMed PMID: 19544558.	Cancer not outcome
34.	De Benedetta G, Bolognini I, D'Ovidio S, Pinto A. Cancer and anorexia nervosa in the adolescence: a family-based systemic intervention. Int J Family Med. 2011;2011:769869. doi: 10.1155/2011/769869. Epub 2011 Aug 7. PubMed PMID: 22295193	Study design
35.	Tabarés-Seisdedos R, Dumont N, Baudot A, Valderas JM, Climent J, Valencia A, Crespo-Facorro B, Vieta E, Gómez-Beneyto M, Martínez S, Rubenstein JL. No paradox, no progress: inverse cancer comorbidity in people with other complex diseases. Lancet Oncol. 2011 Jun;12(6):604-8. doi: 10.1016/S1470-2045(11)70041-9. Epub 2011 Apr 15. Review. PubMed PMID: 21498115.	Study design

© 2019 Catalá-López F et al. *JAMA Network Open.*

36. Landi F, Liperoti R, Lattanzio F, Russo A, Tosato M, Barillaro C, Bernabei R, Onder G. Effects of anorexia on mortality among older adults receiving home care: an observation study. J Nutr Health Aging. 2012 Jan;16(1):79-83. PubMed PMID: 22238005.	Cancer not outcome
37. Franko DL, Keshaviah A, Eddy KT, Krishna M, Davis MC, Keel PK, Herzog DB. A longitudinal investigation of mortality in anorexia nervosa and bulimia nervosa. Am J Psychiatry. 2013 Aug;170(8):917-25. doi: 10.1176/appi.ajp.2013.12070868. PubMed PMID: 23771148	Cancer not outcome
38. Ajetunmobi O, Taylor M, Stockton D, Wood R. Early death in those previously hospitalised for mental healthcare in Scotland: a nationwide cohort study, 1986-2010. BMJ Open. 2013 Jul 30;3(7). pii: e002768. doi: 10.1136/bmjopen-2013-002768. PubMed PMID: 23901025	Data not abstractabl e
39. Hoang U, Goldacre M, James A. Mortality following hospital discharge with a diagnosis of eating disorder: national record linkage study, England, 2001-2009. Int J Eat Disord. 2014 Jul;47(5):507-15. doi: 10.1002/eat.22249. PubMed PMID: 24599787.	Cancer not outcome
40. Winkler LA, Bilenberg N, Hørder K, Støving RK. Does specialization of treatment influence mortality in eating disorders?--A comparison of two retrospective cohorts. Psychiatry Res. 2015 Dec 15;230(2):165-71. doi:10.1016/j.psychres.2015.08.032. PubMed PMID: 26391650.	Cancer not outcome
41. Suokas J, Gissler M, Haukka J, Linna M, Raevuori A, Suvisaari J. [Outcome of eating disorder patients treated in tertiary care]. Duodecim. 2015;131(8):744-52. Finnish. PubMed PMID: 26237890.	Cancer not outcome
42. Zerwas S, Larsen JT, Petersen L, Thornton LM, Mortensen PB, Bulik CM. The incidence of eating disorders in a Danish register study: Associations with suicide risk and mortality. J Psychiatr Res. 2015 Jun;65:16-22. doi: 10.1016/j.jpsychires.2015.03.003. PubMed PMID: 25958083	Cancer not outcome
43. Javaras KN, Runfola CD, Thornton LM, Agerbo E, Birgegård A, Norring C, Yao S, Råstam M, Larsson H, Lichtenstein P, Bulik CM. Sex- and age-specific incidence of healthcare-register-recorded eating disorders in the complete swedish 1979-2001 birth cohort. Int J Eat Disord. 2015 Dec;48(8):1070-81. doi: 10.1002/eat.22467. PubMed PMID: 26769444	Cancer not outcome
44. De Filippo E, Marra M, Alfinito F, Di Guglielmo ML, Majorano P, Cerciello G, De Caprio C, Contaldo F, Pasanisi F. Hematological complications in anorexia nervosa. Eur J Clin Nutr. 2016 Nov;70(11):1305-1308. doi: 10.1038/ejcn.2016.115. Epub 2016 Jul 20. PubMed PMID: 27436150	Study design
45. Fichter MM, Quadflieg N. Mortality in eating disorders - results of a large prospective clinical longitudinal study. Int J Eat Disord. 2016 Apr;49(4):391-401. doi: 10.1002/eat.22501. PubMed PMID: 26767344.	Study design
46. Kask J, Ekselius L, Brandt L, Kollia N, Ekbom A, Papadopoulos FC. Mortality in Women With Anorexia Nervosa: The Role of Comorbid Psychiatric Disorders. Psychosom Med. 2016 Oct;78(8):910-919. PubMed PMID: 27136502.	Cancer not outcome
47. Kask J, Ramklint M, Kolia N, Panagiotakos D, Ekbom A, Ekselius L, Papadopoulos FC. Anorexia nervosa in males: excess mortality and psychiatric co-morbidity in 609 Swedish in-patients. Psychol Med. 2017 Feb 6:1-11. doi: 10.1017/S0033291717000034. [Epub ahead of print] PubMed PMID: 28162109.	Cancer not outcome
48. Catalá-López F, Hutton B, Driver JA, Page MJ, Ridao M, Valderas JM, Alonso-Arroyo A, Forés Martos J, Martínez S, Gènova-Maleras R, Macías-Saint-Gerons D, Crespo-Facorro B, Vieta E, Valencia A, Tabarés-Seisdedos R. Cancer and central nervous system disorders: protocol for an umbrella review of systematic reviews and updated meta-analyses of observational studies. Syst Rev. 2017 Apr 4;6(1):69. doi: 10.1186/s13643-017-0466-y. PubMed PMID: 28376926.	Study design (protocol)
49. Catalá-López F, Hutton B, Driver JA, Ridao M, Valderas JM, Gènova-Maleras R, Forés-Martos J, Alonso-Arroyo A, Saint-Gerons DM, Vieta E, Valencia A, Tabarés-Seisdedos R. Anorexia nervosa and cancer: a protocol for a systematic review and	Study design (protocol)

© 2019 Catalá-López F et al. *JAMA Network Open.*

meta-analysis of observational studies. Syst Rev. 2017;6(1):137. doi: 10.1186/s13643-017-0540-5. PubMed PMID: 28693568.	

© 2019 Catalá-López F et al. *JAMA Network Open.*

eTable 5. Results of Individual Studies

Author, year	Study design (country) Setting, coverage	Number of participants (sex)	Endpoint measure	Adjustment for confounding factors	Point estimates (95% confidence intervals)
Mellemkjaer, 2001[12]	Cohort, retrospective (Denmark) Inpatient, population-based	2337 AN participants (92.0% women)	SIR	Age, sex and calendar year	All-cancer, incidence (both): 0.82 (0.55-1.23)[a]<br>All-cancer, incidence (men): 1.30 (0.20-4.70)<br>All-cancer, incidence (women): 0.80 (0.52-1.18)<br>Brain and central nervous system, incidence (both): 5.71 (1.32-24.71)[a]<br>Brain and central nervous system, incidence (men): 12.50 (1.50-45.00)<br>Brain and central nervous system, incidence (women): 0.60 (0.00-3.20)<br>Lip, oral, incidence (women): 3.20 (0.10-17.40)<br>Esophagus, incidence (women): 20.0 (0.50-111.0)<br>Stomach, incidence (women): 3.80 (0.10-21.40)<br>Pancreas, incidence (women): 4.40 (0.10-24.20)<br>Lung, incidence (women): 2.20 (0.50-6.40)<br>Breast, incidence (women): 0.80 (0.30-1.70)<br>Cervix, incidence (women): 0.60 (0.10-2.00)<br>Bladder, incidence (women): 1.60 (0.01-8.70)<br>Melanoma of skin, incidence (women): 0.60 (0.20-1.50)<br>Thyroid, incidence (women): 1.80 (0.01-10.00)<br>Non-Hodgkin lymphoma, incidence (women): 3.00 (0.40-10.80)
Korndörfer, 2003[30]	Cohort, retrospective (United States) Community, population-based	208 AN participants (92.8% women)	SMR	Age and sex	All-cancer mortality (both): 0.39 (0.08-1.13)
Michels, 2004[13]	Cohort, retrospective (Sweden) Inpatient, population-based	7303 AN participants (100% women)	SIR	Age and calendar year	All-cancer, incidence (women): 0.92 (0.69-1.21)<br>Breast, incidence (women): 0.47 (0.19-0.97)<br>-<br>Additional analysis (by parity status)<br>All-cancer, incidence (parous women): 0.66 (0.38-1.07)<br>All-cancer, incidence (nulliparous women): 1.12 (0.78-1.55)<br>Breast, incidence (parous women): 0.24 (0.03-0.87)<br>Breast, incidence (nulliparous women): 0.77 (0.25-1.79)<br>-<br>Additional analysis (age at anorexia nervosa):<br>Breast, incidence (<20 y women): 0.02 (0.01-1.39)[a]<br>Breast, incidence (20-29 y women): 0.62 (0.17-1.60)<br>Breast, incidence (30-39 y women): 0.53 (0.11-1.54)<br>Breast, incidence (20-39 y women): 0.58 (0.25-1.36)[a]

© 2019 Catalá-López F et al. *JAMA Network Open.*

Karaminis, 2014[23-25]	Cohort, retrospective (Sweden) Inpatient, population-based	6009 AN participants (100% women)	SIR<br>SMR	Age and calendar year	All-cancer, incidence (women): 1.10 (0.80-1.30)<br>All-cancer mortality (women): 2.00 (1.30-2.90)<br>Lip, oral, incidence (both): 1.00 (0.01-5.40)<br>Stomach, incidence (both): 1.40 (0.60-2.70)[b]<br>Stomach, mortality (both): 2.30 (0.80-5.00)[b]<br>Lung, incidence (both): 2.70 (1.00-6.00)<br>Lung, mortality (both): 2.50 (0.70-6.30)<br>Bone and articular cartilage, mortality (both): 7.40 (0.20-41.0)<br>Melanoma of skin, incidence (both): 1.40 (0.70-2.50)<br>Melanoma of skin, mortality (both): 9.60 (3.10-22.0)<br>Other skin cancer, incidence (both): 1.80 (0.20-6.60)<br>Breast, incidence (women): 0.70 (0.40-1.10)<br>Breast, mortality (women): 0.50 (0.10-1.90)<br>Brain and central nervous system, incidence (both): 0.90 (0.30-2.00)<br>Thyroid, incidence (both): 1.10 (0.40-2.40)<br>Lymphoid, hematopoietic, incidence (both): 3.40 (0.90-8.60)<br>Lymphoid, hematopoietic, mortality (both): 1.40 (0.20-5.00)<br>Female genital organs, incidence (women): 0.90 (0.50-1.60)<br>Female genital organs, mortality (women): 2.70 (1.00-5.90)<br>-<br>Additional analysis (by parity status):<br>Breast, incidence (parous women): 0.30 (0.10-0.90)<br>Breast, incidence (nulliparous women): 0.90 (0.40-1.50)<br>-<br>Additional analysis (age at anorexia nervosa):<br>Breast, incidence (10-24 y women): 0.40 (0.10-0.90)<br>Breast, incidence (25-50 y women): 0.80 (0.40-1.50)
Brewster, 2015[31]	Cohort, retrospective (United Kingdom) Inpatient, population-based	2138 AN participants (60.5% women)	SIR	Age, sex, calendar year, and deprivation	Esophagus, incidence (both): 9.96 (4.00-20.51)<br>Stomach, incidence (both): 1.02 (0.03-5.69)<br>Lung, incidence (both): 1.22 (0.49-2.51)
Mellemkjaer, 2015[29,32]	Cohort, retrospective (Denmark, Finland and Sweden) Inpatient, population-based	24332 AN participants (93.1% women)	IRR<br>HR	Age, sex, calendar year, and country	All-cancer, incidence (both): 0.98 (0.88-1.08)<br>All-cancer, incidence (men): 1.08 (0.71-1.66)<br>All-cancer, incidence (women): 0.97 (0.88-1.08)<br>Esophagus, incidence (women): 5.10 (1.80-14.60)<br>Colorectal, incidence (both): 1.15 (0.77-1.71)<br>Colorectal, incidence (women): 1.20 (0.80-1.80)<br>Colorectal, incidence (men): 0.50 (0.10-3.70)<br>Pancreas, incidence (both): 1.98 (0.90-4.34)<br>Pancreas, incidence (women): 1.80 (0.80-4.30)<br>Pancreas, incidence (men): 3.80 (0.40-34.30)<br>Breast, incidence (women): 0.60 (0.50-0.80)

© 2019 Catalá-López F et al. *JAMA Network Open.*

					Breast, mortality (women): 2.10 (1.30-3.60)<br>Uterus, incidence (women): 0.70 (0.30-1.50)<br>Kidney, incidence (both): 1.35 (0.47-3.93)<br>Kidney, incidence (women): 1.00 (0.30-3.30)<br>Kidney, incidence (men): 4.20 (0.40-40.90)<br>Gallbladder, incidence (women): 2.00 (0.40-8.70)<br>Ovary, incidence (women): 0.60 (0.30-1.10)<br>Prostate, incidence (men): 0.50 (0.10-3.40)<br>Thyroid, incidence (women): 0.90 (0.50-1.70)<br>Lymphoid, hematopoietic, incidence (both): 1.23 (0.89-1.69)<br>Lymphoid, hematopoietic, incidence (women): 1.10 (0.80-1.60)<br>Lymphoid, hematopoietic, incidence (men): 2.20 (1.00-5.00)<br>Liver, incidence (women): 5.20 (2.30-12.10)<br>Lung, incidence (both): 1.57 (1.07-2.30)<br>Lung, incidence (women): 1.60 (1.10-2.40)<br>Lung, incidence (men): 0.90 (0.10-6.80)<br>Melanoma of skin, incidence (both): 1.09 (0.80-1.48)<br>Melanoma of skin, incidence (women): 1.10 (0.80-1.50)<br>Melanoma of skin, incidence (men): 0.70 (0.10-5.50)<br>Other skin cancer, incidence (both): 1.10 (0.80-1.50)<br>Other skin cancer, incidence (women): 1.10 (0.80-1.50)<br>Other skin cancer, incidence (men): 1.10 (0.80-1.50)<br>Cervix, incidence (women): 0.70 (0.40-1.00)<br>Testicular, incidence (men): 0.30 (0.01-2.30)<br>Brain and nervous system, incidence (both): 1.34 (0.95-1.89)<br>Brain and nervous system, incidence (women): 1.20 (0.80-1.70)<br>Brain and nervous system, incidence (men): 2.30 (1.00-5.20)<br>-<br>Additional analysis (by parity status):<br>Breast, incidence (parous women): 0.50 (0.40-0.70)<br>Breast, incidence (nulliparous women): 0.70 (0.50-1.00)<br>-<br>Additional analysis (age at anorexia nervosa):<br>Breast, incidence (10-19 y women): 0.70 (0.50-1.10)<br>Breast, incidence (20-50 y women): 0.60 (0.40-0.80)
O'Brien, 2017[33]	Cohort, prospective (United States)<br>Community, multi-center	275 AN participants (100% women)	HR	Age, education and race	Breast, incidence (women): 0.27 (0.07-1.07)

[a] Results were calculated based on the original data using a fixed-effect model (as defined in our study protocol). [b]Digestive organ cancers were computed as stomach cancers. AN: anorexia nervosa.

eTable 6. Methodological Quality Assessment of Included Studies Using Newcastle-Ottawa Scale

© 2019 Catalá-López F et al. *JAMA Network Open.*

NEWCASTLE - OTTAWA QUALITY ASSESSMENT SCALE CASE CONTROL STUDIES

Note: A study can be awarded a maximum of one star for each numbered item within the Selection and Exposure categories. A maximum of two stars can be given for Comparability.

Selection

1) Is the case definition adequate?
- a) yes, with independent validation *
- b) yes, e.g. record linkage or based on self-reports
- c) no description

2) Representativeness of the cases
- a) consecutive or obviously representative series of cases *
- b) potential for selection biases or not stated

3) Selection of Controls
- a) community controls *
- b) hospital controls
- c) no description

4) Definition of Controls
- a) no history of disease (endpoint) *
- b) no description of source

Comparability

1) Comparability of cases and controls on the basis of the design or analysis
- a) study controls for age and sex *
- b) study controls for any additional factor (e.g. medication, smoking, comorbidities) *

Exposure

1) Ascertainment of exposure
- a) secure record (eg surgical records) *
- b) structured interview where blind to case/control status *
- c) interview not blinded to case/control status
- d) written self-report or medical record only
- e) no description

2) Same method of ascertainment for cases and controls
- a) yes *
- b) no

3) Non-Response rate
- a) same rate for both groups *
- b) non-respondents described
- c) rate different and no designation

NEWCASTLE - OTTAWA QUALITY ASSESSMENT SCALE COHORT STUDIES

Note: A study can be awarded a maximum of one star for each numbered item within the Selection and Outcome categories. A maximum of two stars can be given for Comparability

Selection

1) Representativeness of the exposed cohort
- a) truly representative of the average in the community *
- b) somewhat representative of the average in the community *
- c) selected group of users (e.g. nurses, volunteers)
- d) no description of the derivation of the cohort

2) Selection of the non-exposed cohort
- a) drawn from the same community as the exposed cohort *
- b) drawn from a different source
- c) no description of the derivation of the non-exposed cohort

3) Ascertainment of exposure
- a) secure record (e.g. surgical records) *
- b) structured interview *
- c) written self-report
- d) no description

4) Demonstration that outcome of interest was not present at start of study
- a) yes *
- b) no

Comparability

1) Comparability of cohorts on the basis of the design or analysis
- a) study controls for age and sex *
- b) study controls for any additional factor (e.g. medication, smoking, comorbidities…) *

Outcome

1) Assessment of outcome
- a) independent blind assessment *
- b) record linkage *
- c) self-report
- d) no description

2) Was follow-up long enough for outcomes to occur
- a) yes *
- b) no

3) Adequacy of follow up of cohorts
- a) complete follow up - all subjects accounted for *
- b) subjects lost to follow up unlikely to introduce bias *
- c) follow up rate < 40% (select an adequate %) and/or no description of those lost
- d) no statement

© 2019 Catalá-López F et al. *JAMA Network Open.*

Author, year	1. Selection				2. Comparability	3. Outcome			Total score	Quality assessment – risk of bias rating
	**1.1**	**1.2**	**1.3**	**1.4**	**2.1**	**3.1**	**3.2**	**3.3**		
Mellemkjaer, 2001[12]	*	*	*	*	*	*	*	*	8	Low risk
Korndörfer, 2003[30]	*		*	*	*	*	*	*	7	Low risk
Michels, 2004[13]	*	*	*	*	*	*	*	*	8	Low risk
Karaminis, 2014[23-25]	*	*	*	*	*	*	*	*	8	Low risk
Brewster, 2015[31]	*	*	*	*	**	*	*		8	Low risk
Mellemkjaer, 2015[29,32]	*	*	*	*	*	*	*		7	Low risk
O'Brien, 2017[33]		*		*	*		*	*	5	Moderate risk

Note: Risk of bias rating: 0-3 high risk of bias (low quality), 4-6 moderate risk of bias (moderate quality), 7-9 low risk of bias (high quality).

© 2019 Catalá-López F et al. *JAMA Network Open.*

eTable 7. Grading Certainty or Credibility of Evidence for Summary Estimates

Global Burden of Disease (GBD)/World Cancer Research Fund (WCRF)/American Institute for Cancer Research (AICR) criteria and GRADE system emphasize certainty (or credibility) of evidence based on randomized controlled data. For many risks in the biomedical literature (such as disease comorbidity), we will never have randomized controlled trials, and to restrict the assessment of exposures (or risks factors) to only those with trial evidence would lead us to ignore some of the most important determinants of health. Randomized controlled trials are unavailable for our research question. It is for this reason that we have used modified criteria of "convincing/high certainty" or "probable/moderate certainty" evidence for causality. If well designed and reported observational cohort studies form the evidence base the certainty rating starts with high/convincing evidence. If well designed and reported case-control studies form the evidence base the rating starts with moderate/probable evidence. Because the GRADE system does not yet have a scale for assessing non-interventional observational studies, we used a modified version to describe the validity and trustability of the evidence we presented in each meta-analysis. In brief, we rated the evidence as "convincing or high" when we are highly confident that the true effect lies close to that estimated. For example, evidence is judged as "convincing or high" if all of the following apply:<br>• There are multiple cohort studies included in the analyses with no major limitations (low risk of bias according to NOS scale)<br>• Summary effect estimate P value < 10^{-6}<br>• The is little variation between studies (I^2 < 50%)<br>• The summary estimate has a narrow confidence interval (95% predictive interval excluding null value)
We rated the evidence as "probable or moderate" when we consider the true effect is likely to be close to the estimate of the effect, but there is a possibility that it is substantially different. For example, evidence might be judged as "probable or moderate" if any of the following applies:<br>• There are only few studies and some have limitations but not major flaws (low/moderate risk of bias according to NOS scale)<br>• Summary effect estimate P value < 10^{-3}<br>• There is some variation between studies (I^2 = 50-70%)<br>• The 95% predictive interval is wide
Finally, we rated the evidence to be "low (limited, not conclusive or unlikely)" when the true effect may be substantillay different from the estimate of its effect. "Low/limited-suggestive evidence" represents too limited evidence to conclude on a probable or convincing causal association, but where there is evidence suggestive of a direction of effect. "Low/limited-not conclusive evidence" consists of information that is so limited that no firm conclusion can be made for several reasons (e.g., the evidence might be limited by the amount of evidence in terms of the number of studies available, by inconsistency of direction of effect, by poor quality of studies, or by any combination of these factors). For example, evidence might be judged as "low quality" if any of the following apply:<br>• There is only one study or studies have major methological flaws (high risk of bias according to NOS scale)<br>• Summary effect estimate P value < 0.05<br>• There is important variation between study results (I^2 > 70%)<br>• The confidence interval of the summary estimate of the effect is very wide (95% predictive interval including null value or 95% predictive interval was inestimable e.g. less than 3 studies)<br>• Small study effects (sometimes called "publication bias")

Certainty of the evidence and reasons for each outcome of interest.

Outcomes of interest	Certainty of the evidence and reason
**Primary outcome of all cancer**	
All cancer, incidence both sexes	◯◯<br>Low confidence (risk unlikely)<br>Downgraded: (-1) only few studies (n=2), (-1) 95% prediction interval inestimable
All cancer, incidence women	◯◯◯<br>Moderate confidence (risk unlikely)<br>Downgraded: (-1) 95% prediction interval wide

© 2019 Catalá-López F et al. *JAMA Network Open.*

All cancer, incidence men	◯◯<br>Low confidence (limited-not conclusive)<br>Downgraded: (-1) only few studies (n=2), (-1) 95% prediction interval inestimable
All cancer, mortality both sexes	◯◯<br>Low confidence (limited-not conclusive)<br>Downgraded: (-1) only one study, (-1) 95% prediction interval inestimable
All cancer, mortality women	◯◯<br>Low confidence (limited-not conclusive)<br>Downgraded: (-1) only one study, (-1) 95% prediction interval inestimable
**Secondary outcome of site-specific cancer**	
Bladder and urinary, incidence women	◯◯<br>Low confidence (limited-not conclusive)<br>Downgraded: (-1) only one study, (-1) 95% prediction interval inestimable
Bone and articular cartilage, mortality women	◯◯<br>Low confidence (limited-not conclusive)<br>Downgraded: (-1) only one study, (-1) 95% prediction interval inestimable
Brain and central nervous system, incidence both sexes	◯◯<br>Low confidence (limited-not conclusive)<br>Downgraded: (-1) only few studies (n=2), (-1) 95% prediction interval inestimable
Brain and central nervous system, incidence women	◯◯<br>Low confidence (limited-not conclusive)<br>Downgraded: (-2) 95% prediction interval very wide
Brain and central nervous system, incidence men	◯◯<br>Low confidence (limited-not conclusive)<br>Downgraded: (-1) only few studies (n=2), (-1) 95% prediction interval inestimable
Breast, incidence women	◯◯◯◯<br>High confidence (convincing)
Breast, mortality women	◯◯<br>Low confidence (limited-not conclusive)<br>Downgraded: (-1) only few studies (n=2), (-1) 95% prediction interval inestimable
Cervix, incidence women	◯◯<br>Low confidence (limited-not conclusive)<br>Downgraded: (-1) only few studies (n=2), (-1) 95% prediction interval inestimable
Colon and rectum, incidence both sexes	◯◯<br>Low confidence (limited-not conclusive)<br>Downgraded: (-1) only one study, (-1) 95% prediction interval inestimable
Colon and rectum, incidence women	◯◯<br>Low confidence (limited-not conclusive)<br>Downgraded: (-1) only one study, (-1) 95% prediction interval inestimable
Colon and rectum, incidence men	◯◯<br>Low confidence (limited-not conclusive)<br>Downgraded: (-1) only one study, (-1) 95% prediction interval inestimable
Esophagus, incidence both sexes	◯◯<br>Low confidence (limited-not conclusive)<br>Downgraded: (-1) only one study, (-1) 95% prediction interval inestimable
Esophagus, incidence women	◯◯<br>Low confidence (limited-not conclusive)<br>Downgraded: (-1) only few studies (n=2), (-1) 95% prediction interval inestimable
Gallbladder, incidence women	◯◯<br>Low confidence (limited-not conclusive)<br>Downgraded: (-1) only one study, (-1) 95% prediction interval inestimable
Kidney, incidence both sexes	◯◯<br>Low confidence (limited-not conclusive)<br>Downgraded: (-1) only one study, (-1) 95% prediction interval inestimable
Kidney, incidence women	◯◯<br>Low confidence (limited-not conclusive)<br>Downgraded: (-1) only one study, (-1) 95% prediction interval inestimable
Kidney, incidence men	◯◯<br>Low confidence (limited-not conclusive)<br>Downgraded: (-1) only one study, (-1) 95% prediction interval inestimable
Lip and oral cavity, incidence women	◯◯<br>Low confidence (limited-not conclusive)<br>Downgraded: (-1) only few studies (n=2), (-1) 95% prediction interval inestimable
Liver, incidence women	◯◯<br>Low confidence (limited-not conclusive)<br>Downgraded: (-1) only one study, (-1) 95% prediction interval inestimable
Lung, incidence both sexes	◯◯<br>Low confidence (limited-suggestive)<br>Downgraded: (-1) only few studies (n=2), (-1) 95% prediction interval inestimable

© 2019 Catalá-López F et al. *JAMA Network Open.*

Lung, incidence women	◯◯<br>Low confidence (limited-suggestive)<br>Downgraded: (-2) 95% prediction interval very wide
Lung, incidence men	◯◯<br>Low confidence (limited-not conclusive)<br>Downgraded: (-1) only one study, (-1) 95% prediction interval inestimable
Lung, mortality women	◯◯<br>Low confidence (limited-not conclusive)<br>Downgraded: (-1) only one study, (-1) 95% prediction interval inestimable
Lymphoid and hematopoietic, incidence both sexes	◯◯<br>Low confidence (limited-not conclusive)<br>Downgraded: (-1) only one study, (-1) 95% prediction interval inestimable
Lymphoid and hematopoietic, incidence women	◯◯<br>Low confidence (limited-not conclusive)<br>Downgraded: (-2) 95% prediction interval very wide
Lymphoid and hematopoietic, incidence men	◯◯<br>Low confidence (limited-not conclusive)<br>Downgraded: (-1) only one study, (-1) 95% prediction interval inestimable
Lymphoid and hematopoietic, mortality women	◯◯<br>Low confidence (limited-not conclusive)<br>Downgraded: (-1) only one study, (-1) 95% prediction interval inestimable
Malignant skin melanoma, incidence both sexes	◯◯<br>Low confidence (limited-not conclusive)<br>Downgraded: (-1) only one study, (-1) 95% prediction interval inestimable
Malignant skin melanoma, incidence women	◯◯<br>Low confidence (limited-not conclusive)<br>Downgraded: (-2) 95% prediction interval very wide
Malignant skin melanoma, incidence men	◯◯<br>Low confidence (limited-not conclusive)<br>Downgraded: (-1) only one study, (-1) 95% prediction interval inestimable
Malignant skin melanoma, mortality women	◯◯<br>Low confidence (limited-not conclusive)<br>Downgraded: (-1) only one study, (-1) 95% prediction interval inestimable
Other skin cancer, incidence both sexes	◯◯<br>Low confidence (limited-not conclusive)<br>Downgraded: (-1) only one study, (-1) 95% prediction interval inestimable
Other skin cancer, incidence women	◯◯<br>Low confidence (limited-not conclusive)<br>Downgraded: (-1) only few studies (n=2), (-1) 95% prediction interval inestimable
Other skin cancer, incidence men	◯◯<br>Low confidence (limited-not conclusive)<br>Downgraded: (-1) only one study, (-1) 95% prediction interval inestimable
Ovary, incidence women	◯◯<br>Low confidence (limited-not conclusive)<br>Downgraded: (-1) only one study, (-1) 95% prediction interval inestimable
Pancreas, incidence both sexes	◯◯<br>Low confidence (limited-not conclusive)<br>Downgraded: (-1) only one study, (-1) 95% prediction interval inestimable
Pancreas, incidence women	◯◯<br>Low confidence (limited-not conclusive)<br>Downgraded: (-1) only few studies (n=2), (-1) 95% prediction interval inestimable
Pancreas, incidence men	◯◯<br>Low confidence (limited-not conclusive)<br>Downgraded: (-1) only one study, (-1) 95% prediction interval inestimable
Prostate, incidence men	◯◯<br>Low confidence (limited-not conclusive)<br>Downgraded: (-1) only one study, (-1) 95% prediction interval inestimable
Stomach, incidence both sexes	◯◯<br>Low confidence (limited-not conclusive)<br>Downgraded: (-1) only one study, (-1) 95% prediction interval inestimable
Stomach, incidence women	◯◯<br>Low confidence (limited-not conclusive)<br>Downgraded: (-1) only few studies (n=2), (-1) 95% prediction interval inestimable
Stomach, mortality women	◯◯<br>Low confidence (limited-not conclusive)<br>Downgraded: (-1) only one study, (-1) 95% prediction interval inestimable
Testicular, incidence men	◯◯<br>Low confidence (limited-not conclusive)<br>Downgraded: (-1) only one study, (-1) 95% prediction interval inestimable

© 2019 Catalá-López F et al. *JAMA Network Open.*

Thyroid gland, incidence women	◯◯<br>Low confidence (limited-not conclusive)<br>Downgraded: (-2) 95% prediction interval very wide
Uterus, incidence women	◯◯<br>Low confidence (limited-not conclusive)<br>Downgraded: (-1) only one study, (-1) 95% prediction interval inestimable

© 2019 Catalá-López F et al. *JAMA Network Open.*

eTable 8. Summary Statistics of Risk of Cancer for People With Anorexia Nervosa

**Outcomes of interest**	**No of studies**	**No of participants with AN / cancer cases**	**Pooled RR (95% confidence interval)**	**RR largest study (95% confidence interval)**	**P value effect estimate**	**95% prediction interval**	**I^2 with 95% confidence interval**	**Heterogeneity: P value Cochran's Q**	**Certainty of the evidence**
**Primary outcome of all cancer**									
All cancer, incidence both sexes	2	26669/416	0.97 (0.88 to 1.07)	0.98 (0.88 to 1.08)	0.54	NA	0 (NA)	0.40	◯◯ Low confidence (risk unlikely)
All cancer, incidence women	4	38117/517	0.97 (0.89 to 1.06)	0.97 (0.88 to 1.08)	0.53	0.80 to 1.18	0 (0 to 85)	0.57	◯◯◯ Moderate confidence (risk unlikely)
All cancer, incidence men	2	1864/25	1.09 (0.73 to 1.65)	1.08 (0.71 to 1.66)	0.67	NA	0 (NA)	0.82	◯◯ Low confidence (limited-not conclusive)
All cancer, mortality both sexes	1	208/3	NA	0.39 (0.08 to 1.13)	0.16	NA	NA	NA	◯◯ Low confidence (limited-not conclusive)
All cancer, mortality women	1	6009/29	NA	2.00 (1.30 to 2.90)	0.001	NA	NA	NA	◯◯ Low confidence (limited-not conclusive)
**Secondary outcome of site-specific cancer**									
Bladder and urinary, incidence women	1	2337/1	NA	1.60 (0.01 to 8.70)	0.78	NA	NA	NA	◯◯ Low confidence (limited-not conclusive)
Bone and articular cartilage, mortality women	1	6009/1	NA	7.40 (0.20 to 41.0)	0.14	NA	NA	NA	◯◯ Low confidence (limited-not conclusive)
Brain and central nervous system, incidence both sexes	2	26669/45	2.31 (0.58 to 9.12)	1.34 (0.95 to 1.89)	0.23	NA	72 (NA)	0.06	◯◯ Low confidence (limited-not conclusive)
Brain and central nervous system, incidence women	3	30814/43	1.14 (0.81 to 1.62)	1.20 (0.80 to 1.70)	0.45	0.12 to 10.89	0 (0 to 90)	0.78	◯◯ Low confidence (limited-not conclusive)
Brain and central nervous system, incidence men	2	1864/9	4.52 (0.89 to 22.97)	2.30 (1.00 to 5.20)	0.07	NA	68 (NA)	0.08	◯◯ Low confidence (limited-not conclusive)
Breast, incidence women	5	38392/108	0.60 (0.50 to 0.74)	0.60 (0.50 to 0.80)	<0.0001	0.44 to 0.83	0 (0 to 79)	0.66	◯◯◯◯ High confidence (convincing)
Breast, mortality women	2	28663/18	1.22 (0.31 to 4.77)	2.10 (1.30 to 3.60)	0.78	NA	69 (NA)	0.07	◯◯ Low confidence (limited-not conclusive)

© 2019 Catalá-López F et al. *JAMA Network Open.*

Cervix, incidence women	2	24805/23	0.69 (0.45 to 1.07)	0.70 (0.40 to 1.00)	0.10	NA	0 (NA)	0.85	◯◯ Low confidence (limited-not conclusive)
Colon and rectum, incidence both sexes	1	24332/27	NA	1.15 (0.78 to 1.71)	0.48	NA	NA	NA	◯◯ Low confidence (limited-not conclusive)
Colon and rectum, incidence women	1	22654/26	NA	1.20 (0.80 to 1.80)	0.38	NA	NA	NA	◯◯ Low confidence (limited-not conclusive)
Colon and rectum, incidence men	1	1678/1	NA	0.50 (0.10 to 3.70)	0.45	NA	NA	NA	◯◯ Low confidence (limited-not conclusive)
Esophagus, incidence both sexes	1	2318/7	NA	9.96 (4.00 to 20.51)	<0.0001	NA	NA	NA	◯◯ Low confidence (limited-not conclusive)
Esophagus, incidence women	2	24805/6	6.10 (2.30 to 16.18)	5.10 (1.80 to 14.60)	<0.0001	NA	0 (NA)	0.35	◯◯ Low confidence (limited-not conclusive)
Gallbladder, incidence women	1	22654/1	NA	2.00 (0.40 to 8.70)	0.38	NA	NA	NA	◯◯ Low confidence (limited-not conclusive)
Kidney, incidence both sexes	1	24332/4	NA	1.35 (0.47 to 3.93)	0.58	NA	NA	NA	◯◯ Low confidence (limited-not conclusive)
Kidney, incidence women	1	22654/3	NA	1.00 (0.30 to 3.30)	1.00	NA	NA	NA	◯◯ Low confidence (limited-not conclusive)
Kidney, incidence men	1	1678/1	NA	4.20 (0.40 to 40.90)	0.22	NA	NA	NA	◯◯ Low confidence (limited-not conclusive)
Lip and oral cavity, incidence women	2	8160/2	2.00 (0.27 to 14.73)	3.20 (0.10 to 17.40)	0.49	NA	0 (NA)	0.57	◯◯ Low confidence (limited-not conclusive)
Liver, incidence women	1	22654/8	NA	5.20 (2.30 to 12.10)	<0.0001	NA	NA	NA	◯◯ Low confidence (limited-not conclusive)
Lung, incidence both sexes	2	26650/37	1.50 (1.06 to 2.12)	1.57 (1.07 to 2.30)	0.02	NA	0 (NA)	0.58	◯◯ Low confidence (limited-suggestive)
Lung, incidence women	3	30814/38	1.77 (1.25 to 2.50)	1.60 (1.10 to 2.40)	0.001	0.19 to 16.46	0 (0 to 90)	0.54	◯◯ Low confidence (limited-suggestive)
Lung, incidence men	1	1678/1	NA	0.90 (0.10 to 6.80)	0.92	NA	NA	NA	◯◯

© 2019 Catalá-López F et al. *JAMA Network Open.*

									Low confidence (limited-not conclusive)
Lung, mortality women	1	6009/4	NA	2.50 (0.70 to 6.30)	0.10	NA	NA	NA	◯◯ Low confidence (limited-not conclusive)
Lymphoid and hematopoietic, incidence both sexes	1	24332/37	NA	1.23 (0.89 to 1.69)	0.21	NA	NA	NA	◯◯ Low confidence (limited-not conclusive)
Lymphoid and hematopoietic, incidence women	3	30814/36	1.83 (0.78 to 4.30)	1.10 (0.80 to 1.60)	0.16	0.00 to 17025	57 (0 to 88)	0.10	◯◯ Low confidence (limited-not conclusive)
Lymphoid and hematopoietic, incidence men	1	1678/7	NA	2.20 (1.00 to 5.00)	0.05	NA	NA	NA	◯◯ Low confidence (limited-not conclusive)
Lymphoid and hematopoietic, mortality women	1	6009/2	NA	1.40 (0.20 to 5.00)	0.68	NA	NA	NA	◯◯ Low confidence (limited-not conclusive)
Malignant skin melanoma, incidence both sexes	1	24332/42	NA	1.09 (0.80 to 1.48)	0.58	NA	NA	NA	◯◯ Low confidence (limited-not conclusive)
Malignant skin melanoma, incidence women	3	30814/56	1.10 (0.84 to 1.44)	1.10 (0.80 to 1.50)	0.49	0.19 to 6.39	0 (0 to 90)	0.38	◯◯ Low confidence (limited-not conclusive)
Malignant skin melanoma, incidence men	1	1678/1	NA	0.70 (0.10 to 5.50)	0.73	NA	NA	NA	◯◯ Low confidence (limited-not conclusive)
Malignant skin melanoma, mortality women	1	6009/5	NA	9.60 (3.10 to 22.00)	<0.0001	NA	NA	NA	◯◯ Low confidence (limited-not conclusive)
Other skin cancer, incidence both sexes	1	24332/53	NA	1.10 (0.80 to 1.50)	0.55	NA	NA	NA	◯◯ Low confidence (limited-not conclusive)
Other skin cancer, incidence women	2	28663/50	1.12 (0.82 to 1.52)	1.10 (0.80 to 1.50)	0.48	NA	0 (NA)	0.56	◯◯ Low confidence (limited-not conclusive)
Other skin cancer, incidence men	1	1678/5	NA	1.10 (0.80 to 1.50)	0.55	NA	NA	NA	◯◯ Low confidence (limited-not conclusive)
Ovary, incidence women	1	22654/9	NA	0.60 (0.30 to 1.10)	0.12	NA	NA	NA	◯◯ Low confidence (limited-not conclusive)
Pancreas, incidence both sexes	1	24332/7	NA	1.98 (0.90 to 4.34)	0.09	NA	NA	NA	◯◯

© 2019 Catalá-López F et al. *JAMA Network Open.*

									Low confidence (limited-not conclusive)
Pancreas, incidence women	2	24805/7	1.94 (0.87 to 4.34)	1.80 (0.80 to 4.30)	0.10	NA	0 (NA)	0.54	◯◯ Low confidence (limited-not conclusive)
Pancreas, incidence men	1	1678/1	NA	3.80 (0.41 to 34.30)	0.24	NA	NA	NA	◯◯ Low confidence (limited-not conclusive)
Prostate, incidence men	1	1678/1	NA	0.50 (0.10 to 3.40)	0.44	NA	NA	NA	◯◯ Low confidence (limited-not conclusive)
Stomach, incidence both sexes	1	2318/1	NA	1.02 (0.03 to 5.69)	0.99	NA	NA	NA	◯◯ Low confidence (limited-not conclusive)
Stomach, incidence women	2	8160/9	1.51 (0.73 to 3.11)	1.40 (0.60 to 2.70)	0.27	NA	0 (NA)	0.48	◯◯ Low confidence (limited-not conclusive)
Stomach, mortality women	1	6009/6	NA	2.30 (0.80 to 5.00)	0.07	NA	NA	NA	◯◯ Low confidence (limited-not conclusive)
Testicular, incidence men	1	1678/1	NA	0.30 (0.01 to 2.30)	0.38	NA	NA	NA	◯◯ Low confidence (limited-not conclusive)
Thyroid gland, incidence women	3	30814/18	0.97 (0.59 to 1.60)	0.90 (0.50 to 1.70)	0.91	0.04 to 24.85	0 (0 to 90)	0.88	◯◯ Low confidence (limited-not conclusive)
Uterus, incidence women	1	22654/7	NA	0.70 (0.30 to 1.50)	0.38	NA	NA	NA	◯◯ Low confidence (limited-not conclusive)

AN: anorexia nervosa; RR: risk rate. Note: Predictive intervals were inestimable with less than 3 studies.

© 2019 Catalá-López F et al. *JAMA Network Open.*

eFigure 1. Meta-analysis of Breast Cancer Incidence and Mortality

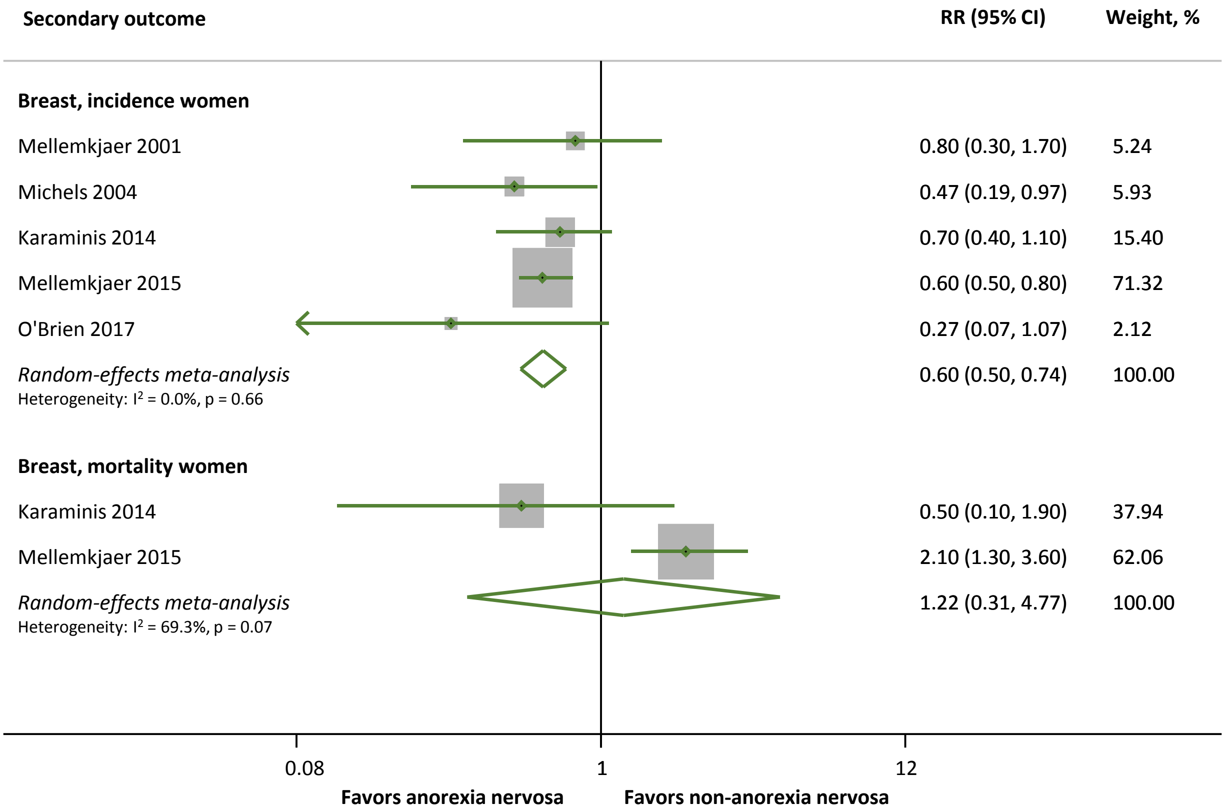

© 2019 Catalá-López F et al. *JAMA Network Open.*

eFigure 2. Meta-analysis of Breast Cancer Incidence and Subgroup Analysis by Parity Status and Age at First Diagnosis of Anorexia Nervosa

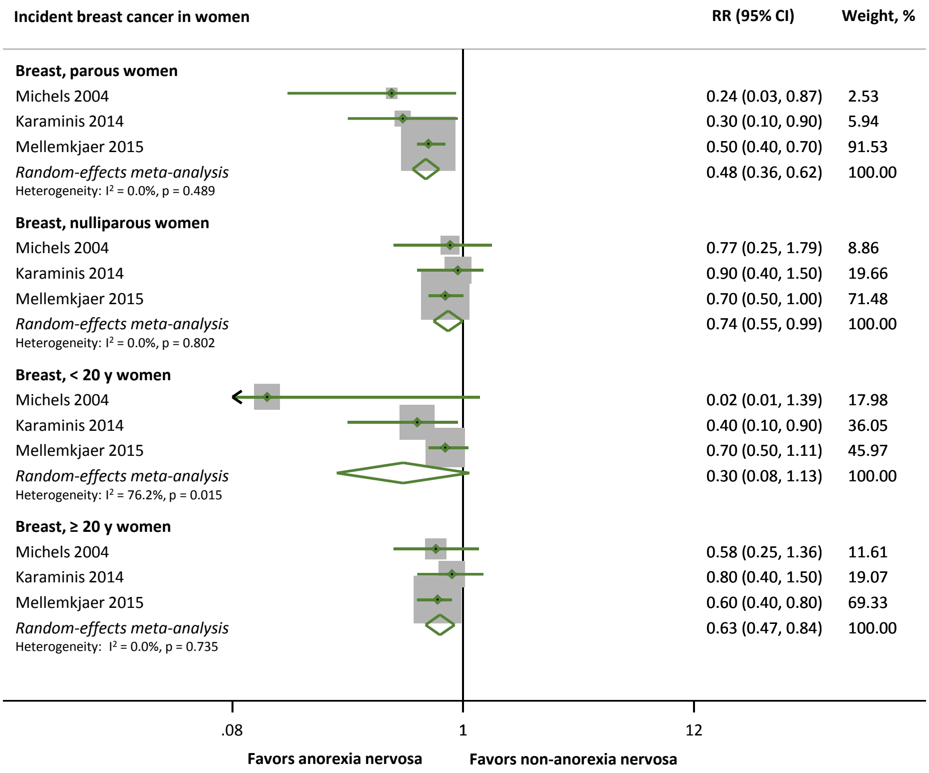

© 2019 Catalá-López F et al. *JAMA Network Open.*

eFigure 3. Meta-analysis of Lung Cancer Incidence

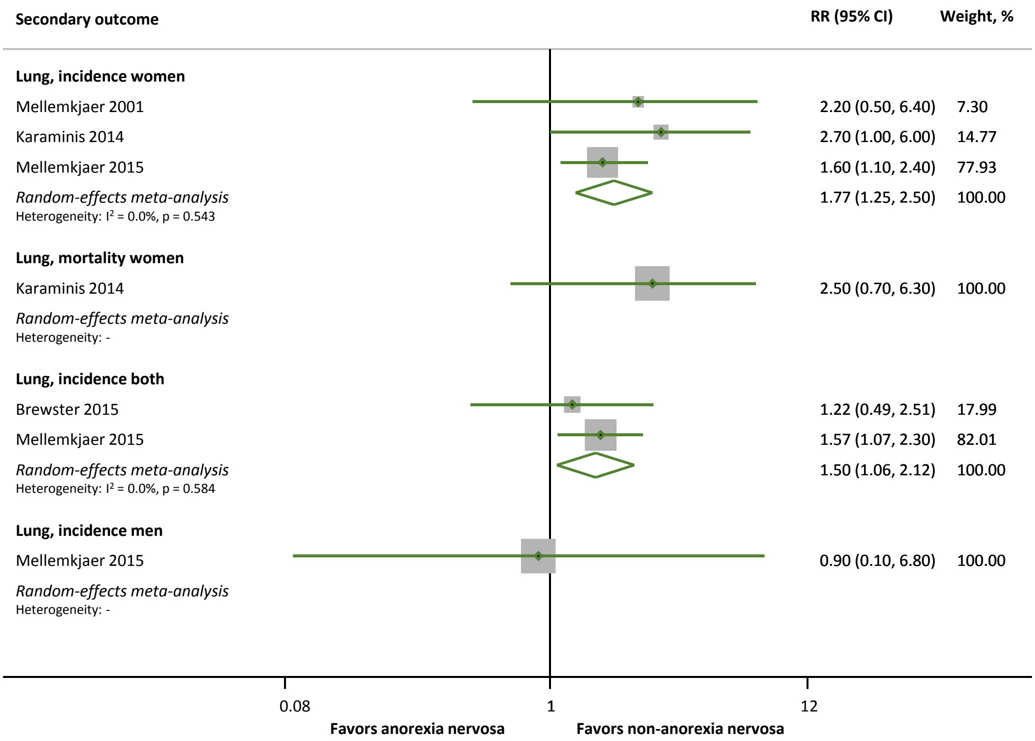

© 2019 Catalá-López F et al. *JAMA Network Open.*

eFigure 4. Meta-analysis of Lymphoid and Hematopoietic Cancer Incidence and Mortality

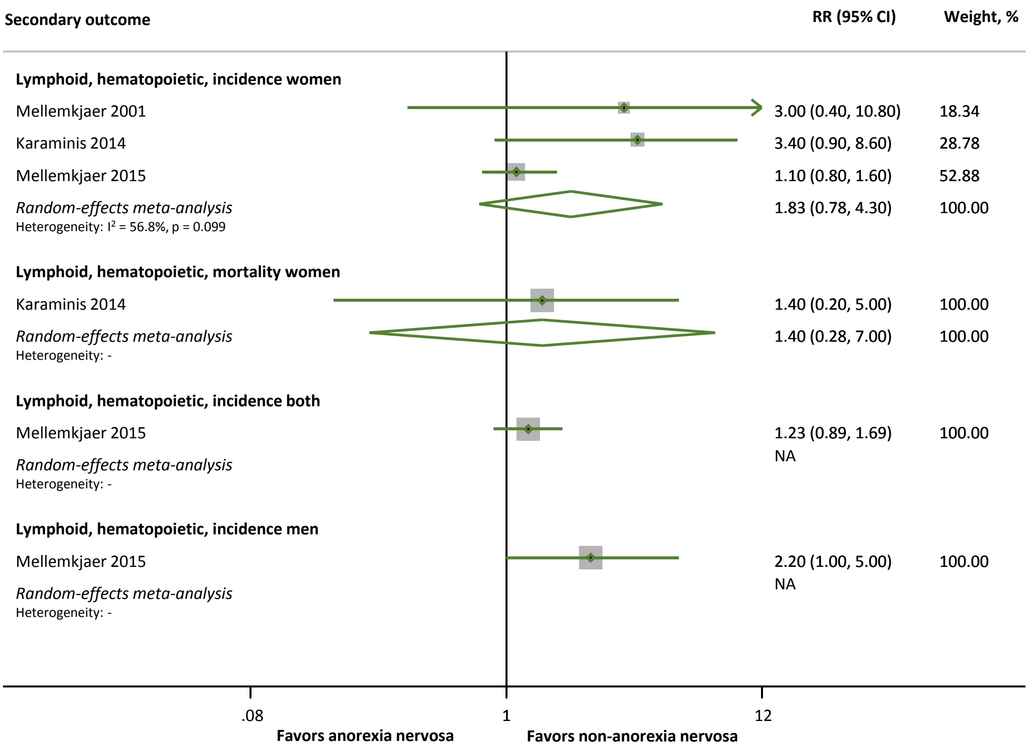

© 2019 Catalá-López F et al. *JAMA Network Open.*

eFigure 5. Meta-analysis of Malignant Skin Melanoma Incidence and Mortality

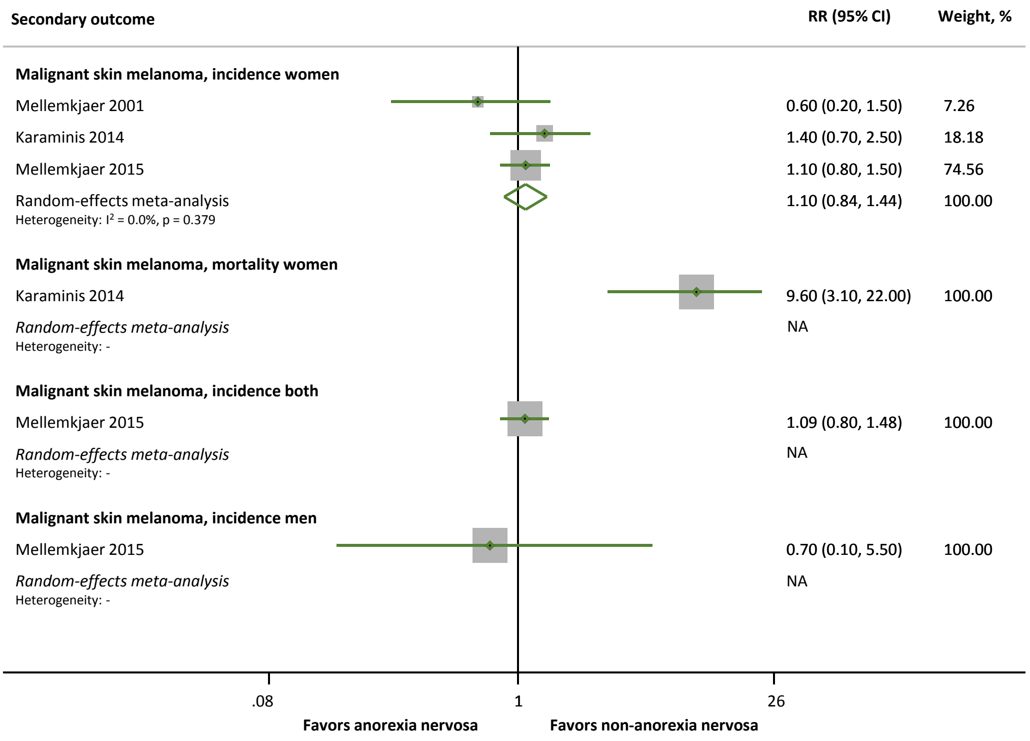

Note: Forest plot generated when ≥ 3 individual studies.

© 2019 Catalá-López F et al. *JAMA Network Open.*

eFigure 6. Meta-analysis of Smoking-Related Cancers

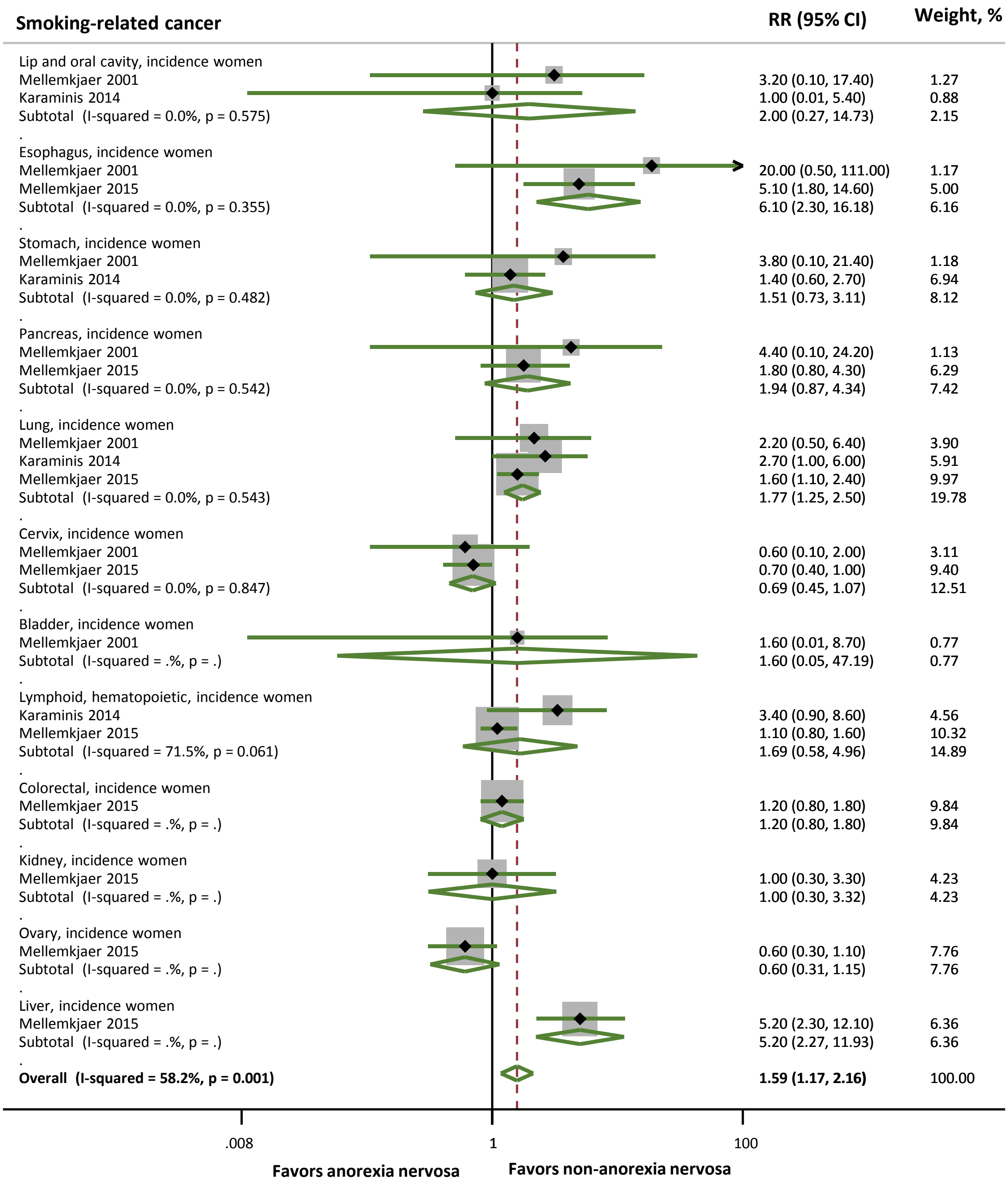

© 2019 Catalá-López F et al. *JAMA Network Open.*

eFigure 7. Meta-analysis of Non–Smoking-Related Cancers

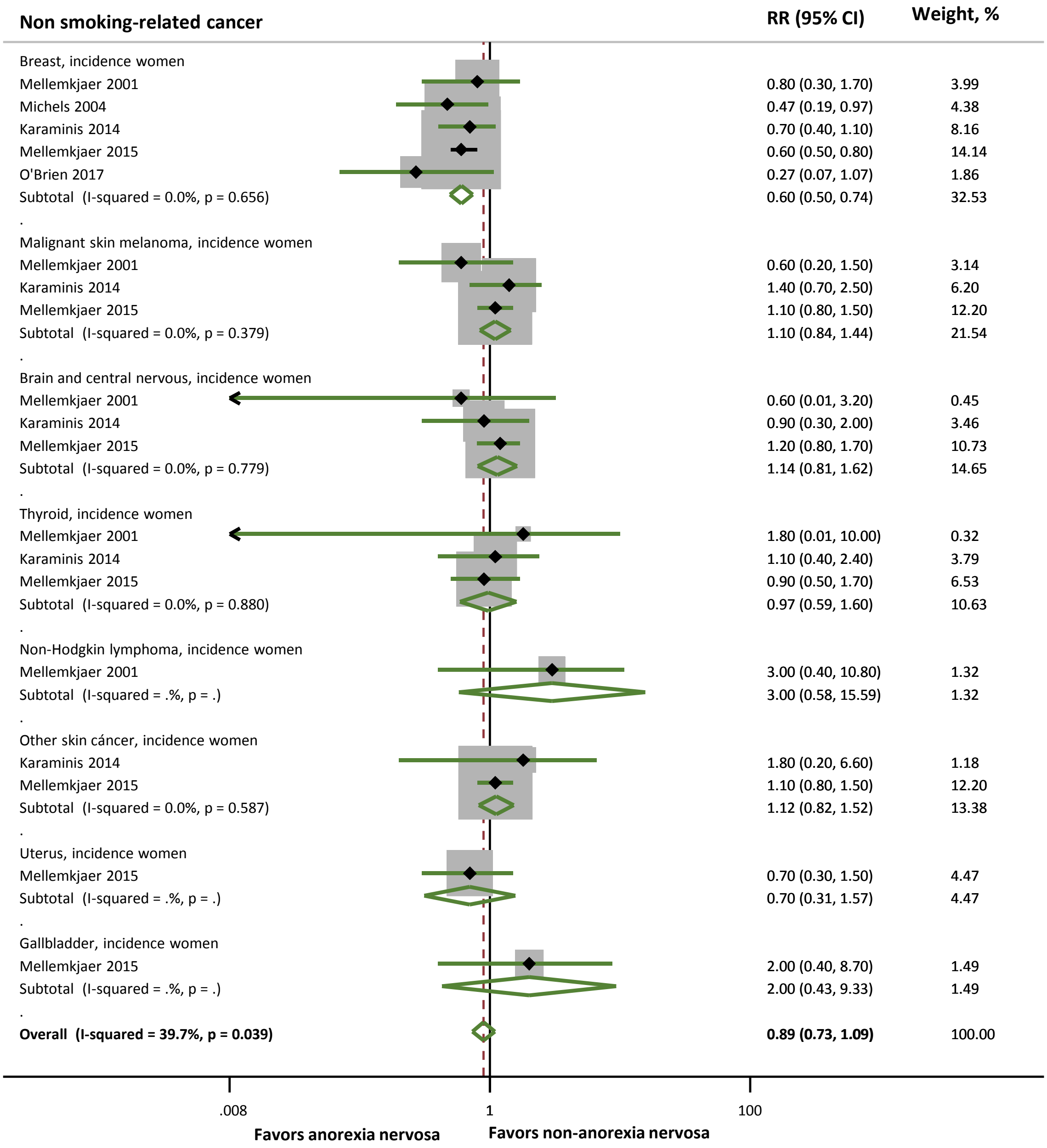

© 2019 Catalá-López F et al. *JAMA Network Open.*

eFigure 8. Meta-analysis of Cancers Occurring in Hormone-Sensitive Tissues

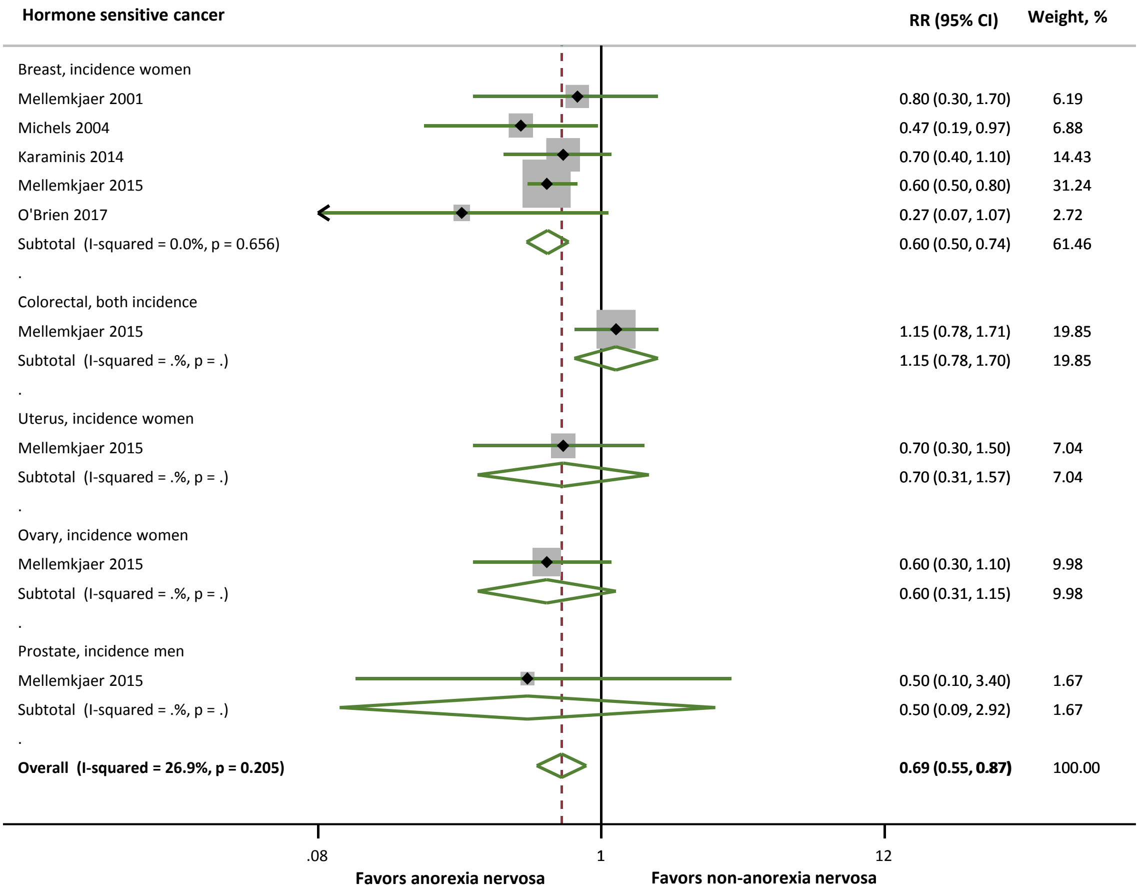

© 2019 Catalá-López F et al. *JAMA Network Open.*

eFigure 9. Sensitivity Analysis

Primary outcome of all cancer incidence and mortality.

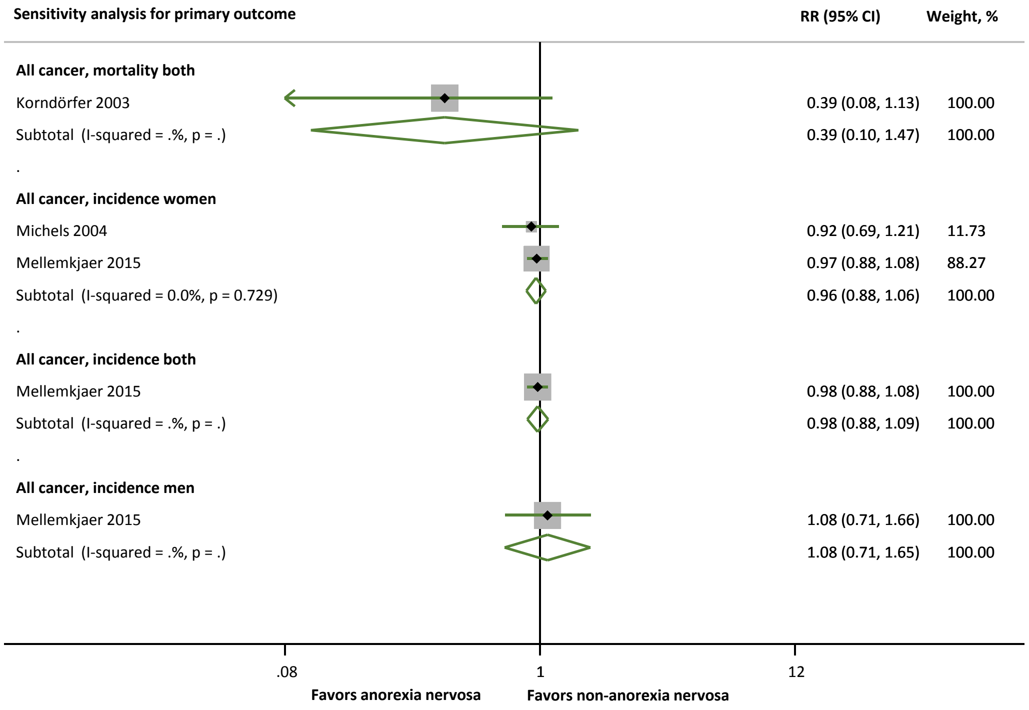

Secondary outcome of breast cancer incidence in women.

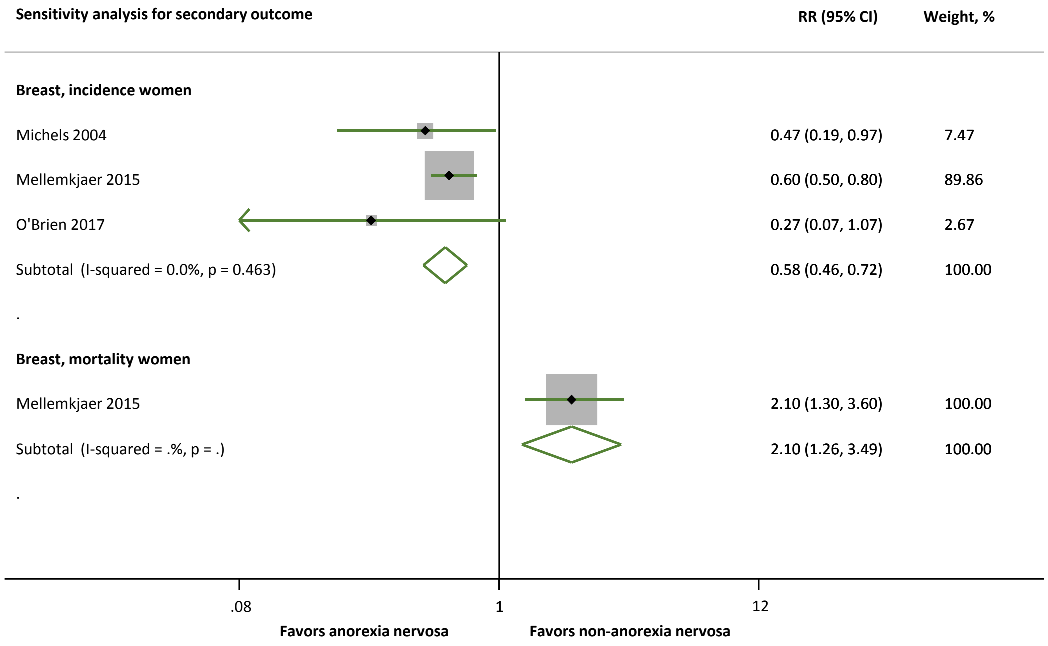

© 2019 Catalá-López F et al. *JAMA Network Open.*
